# Single-cell analysis of StkP activity reveals the temporal dynamics of DivIVA phosphorylation in *Streptococcus pneumoniae*

**DOI:** 10.1128/mbio.01648-26

**Published:** 2026-09-21

**Authors:** Sylvie Manuse, Léo Claude, Pedro M. Alzari, Anne Marie Wehenkel, Christophe Grangeasse

**Affiliations:** 1 Molecular Microbiology and Structural Biochemistry (MMSB), UMR5086, CNRS, Université Lyon 1 236346, Lyon, France; 2 Structural Microbiology Unit, Institut Pasteur, UMR3528, CNRS, Université Paris Cité 27058, Paris, France; 3 Bacterial Cell Cycle Mechanisms Unit, Institut Pasteur, UMR3528, CNRS, Université Paris Cité 27058, Paris, France; University of Florida, Gainesville, Florida, USA

**Keywords:** *Streptococcus pneumoniae*, protein phosphorylation, FHA domain, fluorescent biosensors, StkP, bacterial cell division

## Abstract

Serine/threonine phosphorylation has emerged as a central regulatory mechanism of the bacterial cell cycle. However, the dynamics and temporal selectivity of protein phosphorylation through the cell cycle remain unamenable to existing analytical tools. Although fluorescent phosphorylation biosensors are widely available in single eukaryotic cells, such tools have yet to be adapted for bacteria. To address this gap, we developed an *in vivo* fluorescent biosensor specifically designed to monitor serine/threonine phosphorylation in single bacterial cells. Specifically, this sensor reports protein phosphorylation mediated by the serine/threonine kinase StkP, the main regulator of cell division in *Streptococcus pneumoniae*. We showed that StkP activity is constitutive throughout the cell cycle. However, we observed that StkP-mediated protein phosphorylation is heterogeneous from cell to cell in early stages of the cell cycle. Importantly, our results revealed that the cell division protein DivIVA is found phosphorylated at the cell poles, suggesting an additional regulatory layer controlling its function at this location. Collectively, this work provides original insights into the temporal dynamics of StkP activity and the regulation of the pneumococcal cell cycle by phosphorylation.

**IMPORTANCE:**

How bacterial serine/threonine phosphorylation is dynamically regulated in living single cells remains inaccessible, largely because efficient reporters required to monitor phosphorylation *in vivo* are lacking. In this study, we designed a fluorescent biosensor approach that enables direct visualization of serine/threonine phosphorylation dynamics in single bacterial cells. Using this approach, we revealed that the activity of StkP, the serine/threonine kinase that regulates cell division in *Streptococcus pneumoniae*, is constitutive throughout the cell cycle, yet StkP-mediated protein phosphorylation is unexpectedly heterogeneous across individual cells in the early stages of division. Importantly, our reporter also showed that the phosphorylation of the key cell division protein DivIVA is highly dynamic. Together, these findings provide the conceptual basis to improve our understanding of the role of phosphorylation in pneumococcal cell division and pave the way for investigating phosphorylation dynamics in other bacterial species.

## INTRODUCTION

In bacteria, a large array of protein kinases oversees various cellular processes (1). Among them, serine/threonine protein kinases (STPKs; also known as Hanks-type protein kinases (2)) are pivotal to different stages of the bacterial cell cycle (3, 4). For instance, the three STPKs PrkC, PrkA, and YabT of *Bacillus subtilis* are involved in different aspects of the cell cycle, including cell division, differentiation, and development (sporulation and germination) (5–7). Likewise, phosphorylation of cell division proteins by PkaI is crucial in regulating sporulation of *Streptomyces coelicolor*, while the other STPK AfsK controls cell division and morphogenesis (8). Another notable example concerns the STPKs PknA and PknB of *Mycobacterium tuberculosis,* which are both crucial in regulating cell division and morphogenesis (9–11). Importantly, the Gram-positive pathogen *Streptococcus pneumoniae,* likely the most studied model in this field, encodes a single STPK, the membrane protein StkP, which orchestrates cell division and maintains cell wall homeostasis through phosphorylation of several endogenous substrates (12–14). While phosphoproteomic analyses have identified a large number of StkP substrates (15–18), only the phosphorylation on threonine of four key cell division proteins, MapZ (19, 20), MacP (21), EloR/KhpB/Jag (22–24), and DivIVA (12–14), is consistently detectable by anti-phospho-threonine immunoblotting under normal conditions. MapZ positions the division site at midcell by recruiting the tubulin-like protein FtsZ, which scaffolds the organization of the cell division complex independently of its phosphorylation (19, 20). MacP regulates the class A penicillin-binding protein (PBP), PBP2a, and its phosphorylated form is required to activate PBP2a (21, 25). Regarding EloR (also known as Jag/KhpB), its phosphorylation modulates cell elongation (22–24). Last, DivIVA (26, 27), which controls cell elongation and coordinates septation (19, 28), is regulated by phosphorylation (12, 13). While its phosphorylated form promotes cell wall septal synthesis, its non-phosphorylated form favors cell elongation (19, 29). DivIVA is highly conserved in gram-positive bacteria, especially among Actinobacteria and Firmicutes, where it broadly plays a role in cell division, cell elongation, polar growth, and chromosome segregation (30). To date, no StkP substrates have been detected with anti-phospho-serine antibodies, as phospho-serine proteins detected by immunoblotting were found to be independent of StkP (12).

Although StkP and those substrates are part of the division machinery called the divisome, and therefore localize together at the division septum, the timing and time sequence of StkP-mediated protein phosphorylation events throughout the cell cycle remain enigmatic. Therefore, the precise phases of the cell cycle at which these substrates are phosphorylated remain unknown. This gap is largely due to limitations of current methodologies, which assess protein phosphorylation only in bulk cell populations. Because isolating specific bacterial populations at distinct cell cycle stages is challenging, notably for the pneumococcus in which it has never been reported, it is crucial to develop reporters to track phosphorylation at the single-cell level.

Tracking phosphorylation dynamics in single cells over time and space has been successfully achieved with eukaryotic cells that benefit from a diverse array of phosphorylation biosensors (31, 32). These sensors enable nondestructive and *in vivo* tracking of phosphorylation dynamics. They are thus invaluable for studying phosphorylation heterogeneity in cell populations. In bacteria, a first and unique attempt that relied on a FRET-based sensor showed promising results in *B. subtilis* regarding the activity of the PrkC kinase (33). However, FRET approaches are technically challenging to implement and hardly transposable due to several intrinsic limitations, such as sensor calibration, suboptimal dynamic range, and imaging setups. To overcome these challenges and study the phosphorylation dynamics in single bacterial pneumococcal cells, an attractive strategy consists in using a phosphopeptide-binding domain coupled to a simple fluorescence probe. Such a system could serve as a reporter for StkP-mediated protein phosphorylation, with specificity toward the phosphorylation of its endogenous substrates.

**Fig 1:**
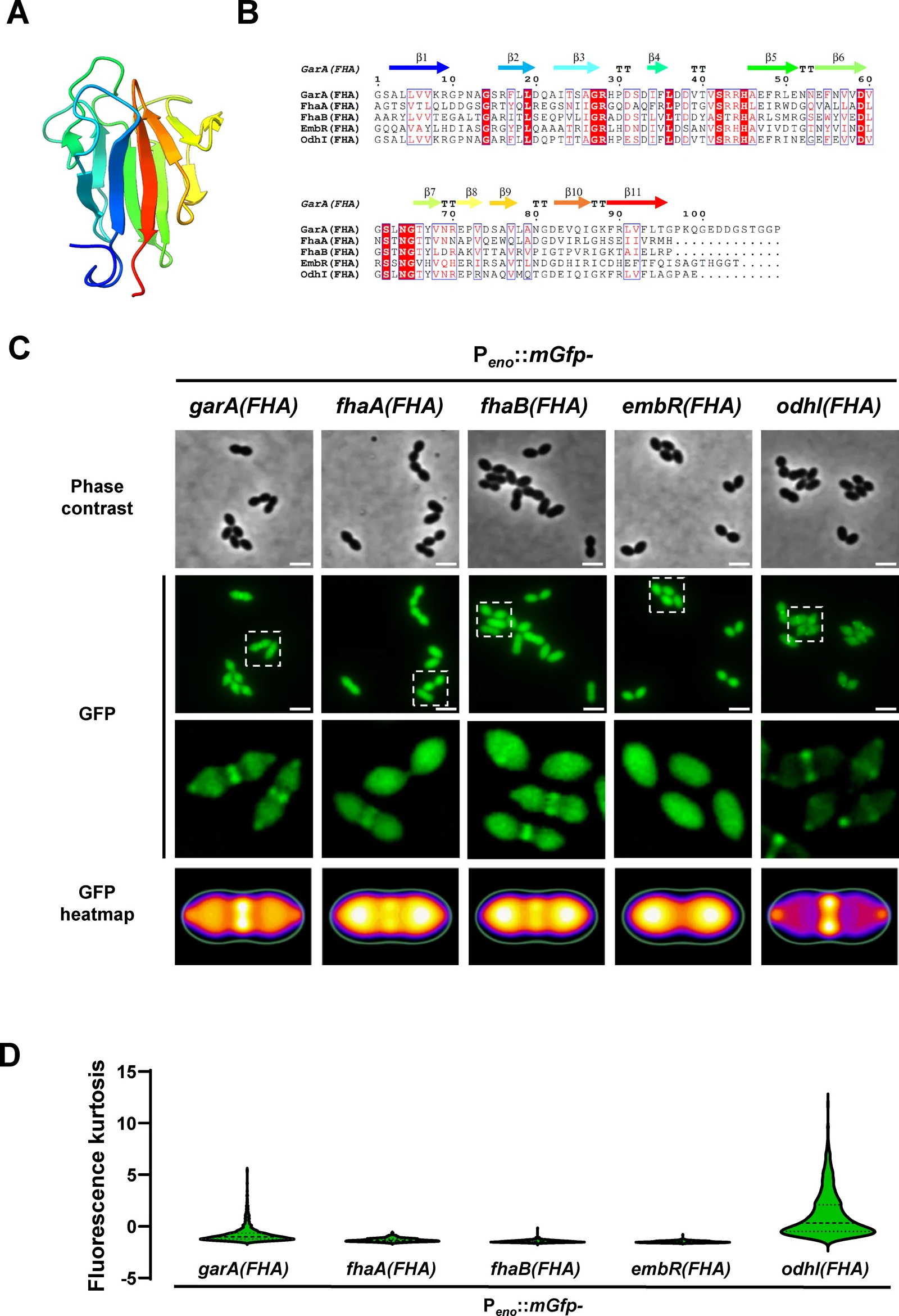
Subcellular localization of FHA-based sensors (**A**) Structural organization of FHA domains. Crystal structure of OdhI FHA’s domain from *Corynebacterium glutamicum* (PDB: 4QCJ) (38) is shown in rainbow color scheme (N-terminus, blue; C-terminus, red), and highlights the typical β-sandwich fold of 11 β-strands found in FHA domains that provides a scaffold for recognizing phosphorylated peptides. (**B**) Sequence alignment of multiple FHA domains. Multiple alignment was performed using ESPript 3.0 (https://espript.ibcp.fr/ESPript/cgi-bin/ESPript.cgi). Identical and similar residues were colored in red. Secondary structures (arrows for β-strands and TT for strict β-turns) were output using 4QCJ and colored-coded to match panel **A**). (**C**) Representative images of exponential cultures of pneumococcal cells expressing the different fluorescent FHA-based sensors. Phase contrast, raw GFP fluorescent signal, and overlays are shown; scale bar, 2 µm. A region of interest, marked with a dashed line, has been magnified for detailed visualization and processed for background correction. Here, raw and processed fluorescence images are shown to highlight the changes made during background correction. Quantification analysis have been performed on processed images, and, for the remainder of the manuscript, only those images have been presented. Heatmaps representing the localization pattern of the different FHA domains are shown; from a total of 900 cells analyzed per strain, only early-constricting cells were selected for illustration purposes. (**D**) Corresponding scatter plots showing the fluorescence kurtosis (the fourth-order moment about the mean) of individual cells, quantified from 900 cells for each strain. Lines indicate the medians and interquartile ranges. Results are representative of independent experiments performed three times.

To date, at least 14 distinct domain types are known to specifically bind phosphoserine (pSer) and phospho-threonine (pThr) residues (34). However, despite the widespread presence of STPKs in bacteria, only one of these phospho-binding domains, the ForkHead-associated (FHA) domain, has been identified in bacteria (35). FHA domains are modular phosphopeptide-binding regions conserved across prokaryotic and eukaryotic organisms, where they regulate cellular processes such as the cell cycle, DNA damage response, and signal transduction (35, 36). Structurally, FHA domains adopt a β-sandwich fold (Fig. 1A), providing a scaffold for recognizing phosphorylated peptides and thus enabling phosphorylation-dependent protein-protein interactions. The high specificity of FHA domains for phosphorylated threonine residues facilitates their role as regulatory hubs in phosphorylation signaling networks (37).

In bacteria, FHA domains are particularly enriched in Actinobacteria, Cyanobacteria, selected Proteobacteria, and found in certain Firmicutes (35). However, most of our knowledge about the structure-function relationship of bacterial FHA domains comes from studies performed in *Mycobacterium tuberculosis*. This bacterium expresses six FHA domain proteins: GarA, EmbR, FhaA, FhaB, Rv1747, and Rv3360 (35). While the roles of Rv1747, a putative transporter of cell wall biosynthesis intermediates (39, 40), and the unknown protein Rv3360 remain to be fully established, the functions of the other four have been more extensively characterized. GarA regulates the tricarboxylic acid (TCA) cycle and glutamate synthesis (41–43), EmbR regulates cell wall arabinosyltransferases linked to ethambutol resistance (44, 45), and FhaA and FhaB are both crucial for cell envelope integrity and morphogenesis (46–50).

Interestingly, *S. pneumoniae* lacks endogenous FHA-containing proteins, which led us to focus on the four characterized FHA domains from *M. tuberculosis* to test their potential as a specific biosensor for StkP substrates. For that, we constructed hybrid proteins composed of the green fluorescent protein (mGFP) fused with various FHA domains and tested their ability to track StkP activity and the phosphorylation of its substrates in single cells. We notably demonstrate that one of these biosensors is efficient at reporting StkP-mediated protein phosphorylation dynamics across the cell cycle by tracking the phosphorylation of the well-known and conserved cell division protein DivIVA. This work thus provides the foundation for new investigations aiming at deciphering the phosphorylation regulatory mechanisms of bacterial cell division.

## RESULTS

### Strategy to construct a sensor for StkP activity

To select the best FHA domains, we focused on the well-characterized mycobacterial proteins GarA, FhaA, FhaB, and EmbR (Fig. 1A and B) and excluded Rv1747 because of its low affinity toward phosphopeptides (51) as well as Rv3360 due to its lack of characterization. Our strategy was then to express the FHA domains of either GarA, FhaA, FhaB, or EmbR fused to mGFP in *S. pneumoniae* and investigate their subcellular localization. Since StkP and its endogenous targets localize at mid-cell, we reasoned that if mGFP-FHA fusions have the ability to interact with the phosphorylated forms of StkP targets, they would localize at mid-cell as well. To achieve this, we expressed ectopically each FHA domain (GarA, FhaA, FhaB, and EmbR) fused to the C-terminus of the mGFP. We used the constitutive promoter and terminator of the enolase gene to ensure consistent expression. These constructs [P*_eno_::mGfp-garA(FHA),* P*_eno_::mGfp-fhaA(FHA*), P*_eno_::mGfp-fhaB(FHA*), P*_eno_::mGfp-embR(FHA*)] were integrated into the genome of *S. pneumoniae* at a neutral locus within the IS*1167* element between *treR* and *amiF* (Fig. S1A and B). All four mGFP fusion proteins were stable and produced at a similar level (Fig. S1C). The fusions were also not harmful, as cells grew in a similar manner as the WT, with only minor autolysis defects and a slight delay in the lag phase for P*_eno_::mGfp-garA(FHA)* (Fig. S1D). Importantly, when looking at the phosphorylation profile using anti-phospho-threonine antibodies, the fusions did not affect StkP activity since the four StkP substrates (MacP, DivIVA, EloR, and MapZ) were all phosphorylated as in the WT strain (Fig. S1C).

Analysis of the subcellular localization revealed distinct patterns for the different mGFP-FHA probes. mGFP-EmbR(FHA) fluorescence was distributed homogeneously in the cytoplasm, suggesting either that it does not bind any phosphorylated proteins or, conversely, that it is promiscuous and can bind more phosphoproteins than the four cell division proteins previously cited (Fig. 1C). On the other hand, the three other mGFP-FHA probes, mGFP-GarA(FHA), mGFP-FhaA(FHA), and mGFP-FhaB(FHA), showed partial septal localization, suggesting interactions with phosphorylated forms of cell division proteins at the division septum (Fig. 1C). Interestingly, mGFP-GarA(FHA) also exhibited a polar localization, likely reflecting its binding to phosphorylated DivIVA, as this protein is the unique cell division protein phosphorylated by StkP known to localize at both midcell and cell poles (14, 27, 52). However, these three probes also show a diffuse localization in the cytoplasm that may potentially be problematic for subsequent analyses. Interestingly, fluorescence kurtosis analysis per cell, which reflects the tailedness of the fluorescence distribution within each cell, showed that mGFP-GarA(FHA) displayed the most spatially restricted localization, associated with the highest fluorescence kurtosis value. This indicates that mGFP-GarA(FHA) was the most suitable among the three biosensors for providing an optimal readout of StkP-mediated phosphorylation of cell division proteins at the division septum (Fig. 1D). To improve this detection, we next considered testing the FHA domain of a GarA homolog. We thus constructed a similar fusion with the FHA domain of OdhI, the homolog of GarA in *Corynebacterium glutamicum*, leading to the P*_eno_::*mGfp-odhI(FHA) construct. Indeed, OdhI shares 78.8% identity with GarA (Fig. 1B), and it is also involved in regulating glutamate synthesis and the TCA cycle (53, 54). As for mGFP-GarA(FHA), mGFP-OdhI(FHA) was stable and did not impact cell growth (Fig. S1C and D). However, strikingly, its localization pattern showed a greater enrichment at the division septum and cell poles, as illustrated by greater fluorescence kurtosis per cell (Fig. 1C and D).

GarA’s and OdhI’s FHA domains can engage in phospho-independent interactions with the 2-oxoglutarate dehydrogenase complex of the TCA cycle (KGD in *M. tuberculosis* and OdhA in *C. glutamicum*) (38, 43). Given that *S. pneumoniae* lacks a complete TCA cycle (55) and, in particular, the 2-oxoglutarate dehydrogenase complex, mGFP-GarA(FHA) and mGFP-OdhI(FHA) would thus represent ideal candidates to track StkP-mediated phosphorylation of cell division proteins without potential detrimental side effects on pneumococcal metabolism. As mGFP-OdhI(FHA) exhibited enhanced septal and polar localization compared to mGFP-GarA(FHA) (Fig. 1C), it was selected as a potential reliable biosensor for StkP-mediated protein phosphorylation in subsequent analyses.

### Validation of mGFP-OdhI(FHA) as a biosensor for StkP activity

**Fig 2:**
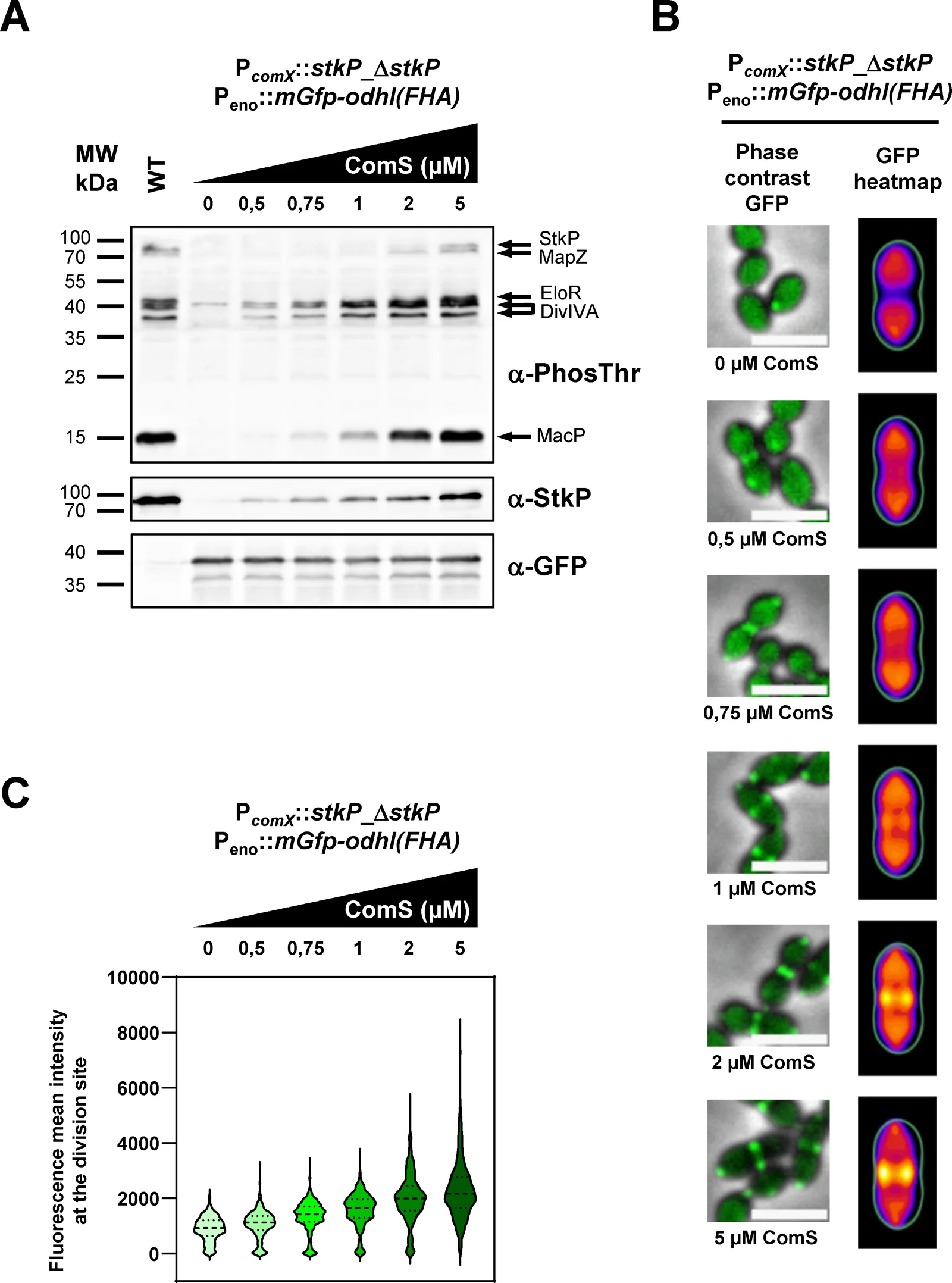
Validation of mGFP-OdhI(FHA) as a sensor for StkP activity. (**A**) Western immunoblots of whole-cell lysates from WT and P*_comX_::stkP*_Δ*stkP*_P*_eno_::mGfp-OdhI(FHA*), grown to exponential phase in the presence of different concentrations of ComS inducer. Extracts were probed with anti-phospho-threonine (α-PhosThr, top panel), anti-StkP (α-StkP, middle panel), and anti-GFP (α-GFP, bottom panel) antibodies. Arrows highlight the phosphorylation signals for the different StkP substrates. (**B**) Representative phase-contrast/fluorescence overlays of exponential-phase cultures of P*_comX_::stkP*_Δ*stkP*_P*_eno_::mGfp-OdhI(FHA*) before analysis in panel **A**; scale bar, 2 µm. Corresponding heatmaps representing the localization pattern of mGFP-OdhI(FHA) are shown on the right; from a total of 450 cells analyzed per strain, only early-constricting cells were selected for illustration purposes. (**C**) Corresponding scatter plots showing the mean fluorescence intensity found at the division site in individual cells, quantified from 450 cells for each strain. Lines indicate the medians and interquartile ranges. Results are representative of independent experiments performed three times.

To confirm that the septal and polar localization signals of the mGFP-OdhI(FHA) probe were dependent on StkP activity, we deleted the *stkP* gene in the P*_eno_::mGfp-odhI(FHA*) strain and then transformed the resulting strain with P*_comX_::stkP* (Δ*stkP*_P*_comX_::stkP*_P*_eno_::mGfp-OdhI(FHA*)) that allows expression of *stkP* under the control of the P*_comX_* inducible promoter (56). This setup allowed us to control StkP levels by adjusting the ComS peptide inducer concentration and to track the localization of mGFP-OdhI(FHA) as a function of *stkP* expression and activity. As expected, fluorescence of mGFP-OdhI(FHA) was diffuse and cytoplasmic in the absence of inducer, reflecting that no phosphoproteins are detected at the division septum and the cell poles in the absence of StkP (Fig. 2A and B). By contrast, addition of increasing concentrations of ComS correlated with increased expression of *stkP* and phosphorylation of its substrates (Fig. 2A), the expression of the probe being unchanged. Importantly, fluorescence also became progressively enriched at the division septum and poles (Fig. 2B). Quantification of the fluorescence intensity at midcell across the different StkP induction levels showed a clear positive correlation with StkP activity, indicating the ability of this probe to function as a readout for StkP-mediated protein phosphorylation (Fig. 2C).

To strengthen these observations, we next analyzed the localization of mGFP-OdhI(FHA) in StkP kinase-dead mutants (Fig. S2A and B). For that, we either deleted *stkP* or introduced the well-characterized inactivating mutation of the catalytic lysine [*stkP*(*K42M*)] (Fig. S2A) (13). We also deleted the PASTA 1, 2, and 3 domains of StkP [*stkP*(Δ*PASTA123*)] that only retains minor kinase activity (Fig. S2A) (57). Analysis of mGFP-OdhI(FHA) fluorescence intensity at midcell in the three strains shows a strong decrease in *stkP*(Δ*PASTA123*), and an absence of any signal in Δ*stkP* and *stkP*(*K42M*) (Fig. S2B). This confirms that the mGFP-OdhI(FHA) signal at mid-cell is a specific readout of StkP activity and phosphorylation of its endogenous targets.

Last, we also assessed the ability of mGFP-OdhI(FHA) to detect changes in phosphorylation under specific treatment. As previously observed by Beilharz et al. (14), the global level of StkP-mediated protein phosphorylation decreases in response to vancomycin. We confirmed this decrease in phosphorylation at the population level using immunoblotting with anti-phospho-threonine antibodies (Fig. S2C). Consistent with this, analysis of mGFP-OdhI(FHA) fluorescence showed a clear decrease of intensity at midcell following vancomycin treatment (Fig. S2D), thus reinforcing the robustness of our approach.

### Identification of StkP substrates interacting with mGFP-OdhI(FHA)

**Fig 3:**
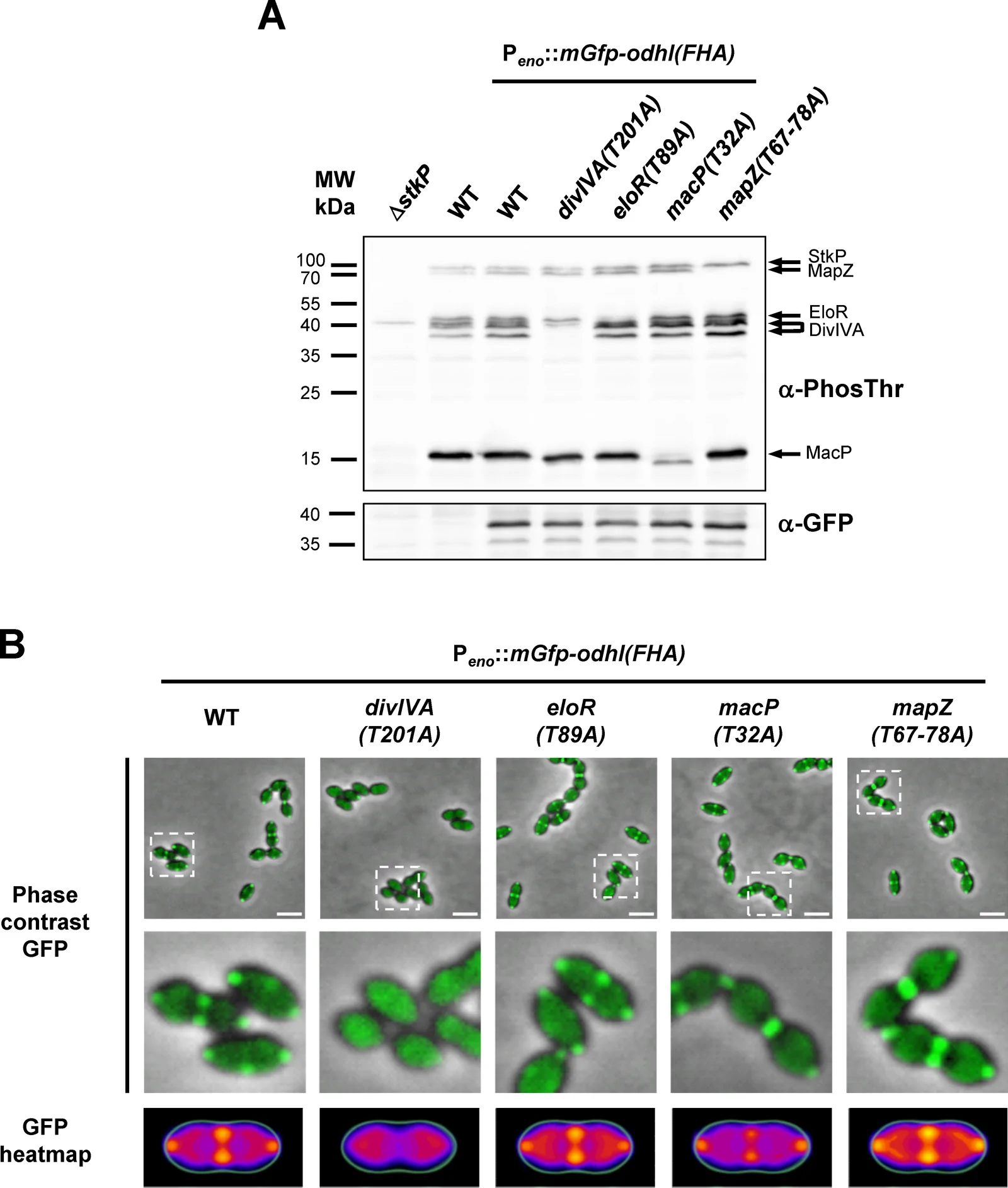
Interaction of mGFP-OdhI(FHA) with phosphorylated DivIVA (**A**) Western immunoblots of whole-cell lysates from phosphoablative mutants expressing P*_eno_::mGfp-OdhI(FHA*), grown to exponential phase. Extracts were probed with anti-phospho-threonine (α-PhosThr, top panel) and anti-GFP (α-GFP, bottom panel) antibodies. Arrows highlight the phosphorylation signals for the different StkP substrates. (**B**) Representative phase-contrast/fluorescence overlays of exponential-phase cultures of phosphoablative mutants expressing P*_eno_::mGfp-OdhI(FHA*) before analysis in (**B**); scale bar, 2 µm. A region of interest, marked with a dashed line, has been magnified for detailed visualization. Corresponding heatmaps representing the localization pattern of mGFP-OdhI(FHA) are shown on the bottom; from a total of 600 cells analyzed per strain, only early-constricting cells were selected for illustration purposes. Results are representative of independent experiments performed three times.

Next, we aimed at deciphering what the phosphorylated cell division proteins which interact with mGFP-OdhI(FHA) are. For that, we expressed the biosensor in strains in which each of the four characterized StkP substrates detectable with anti-phospho-threonine antibodies, DivIVA, EloR, MacP, and MapZ, was substituted for its non-phosphorylatable form: DivIVA(T201A) (13), EloR(T89A) (22–24), MacP(T32A), (21) and MapZ(T67–78A) (19, 20). Anti-phospho-threonine immunoblotting confirmed that the phosphoablative mutations in the four proteins prevent their phosphorylation (Fig. 3A), although an additional and unidentified band could be seen in the *macP* phosphoablative mutant. Besides this, and importantly, all phosphoablative forms did not alter the phosphorylation of the others. Then, we monitored the fluorescence localization pattern of mGFP-OdhI(FHA) to evaluate whether it was altered by the phosphoablative mutations of StkP substrates (Fig. 3B). Fluorescence analysis of mGFP-OdhI(FHA) revealed minor changes of fluorescence intensities in the *eloR(T89A*), *macP(T32A*), and *mapZ(T67–78A*) mutants compared to the WT cells. In addition, we observed that mGFP-OdhI(FHA) localization did not change and stayed at the division septum and the poles. However, we detected a striking difference in the *divIVA(T201A*) strain (Fig. 3A and B). In this strain, septal and polar mGFP-OdhI(FHA) fluorescence was totally abolished. Instead, we noted a diffuse and cytoplasmic fluorescence signal, closely resembling the fluorescence pattern detected in absence of *stkP* expression or activity (Fig. 2; Fig. S2B). Similarly, we observed the same cytoplasmic mGFP-OdhI(FHA) signal in a Δ*divIVA* mutant (Fig. S3). Moreover, GpsB, a positive regulator of StkP activity toward its substrates including DivIVA (13, 58), was also required for proper mGFP-OdhI(FHA) localization, as its fluorescent signal at the division septum was nearly absent and mostly localized in the cytoplasm in a Δ*gpsB* mutant (Fig. S3). Altogether, these findings indicate that the localization of mGFP-OdhI(FHA) to the septum and poles specifically relies on the phosphorylation of DivIVA, establishing it as a selective readout for StkP activity toward DivIVA. Interestingly, the septal and polar fluorescence signals previously observed for the other mGFP-FHA probes mGFP-GarA(FHA), mGFP-FhaA(FHA), and mGFP-FhaB(FHA) (Fig. 1) were also dependent on the phosphorylation of DivIVA (Fig. S4).

Cells expressing *divIVA(T201A*) were previously shown to elongate and to bulge (13). This phenotype was not observed in the present experiments, suggesting that these cell shape defects are dependent on the growth conditions. Supporting this hypothesis, the experiments here were conducted in C + Y medium, while previous observations were made with cells grown in Todd-Hewitt yeast (THY) medium (13). We therefore compared the phenotype of *divIVA(T201A*) cells grown in THY or C + Y media and confirmed that elongation and bulging are exacerbated in THY but not in C + Y (Fig. S5). This observation confirms that the role of DivIVA phosphorylation in the regulation of cell morphology is dependent on the growth conditions.

### Molecular basis of the interaction between phosphorylated DivIVA and mGFP-OdhI(FHA)

**Fig 4:**
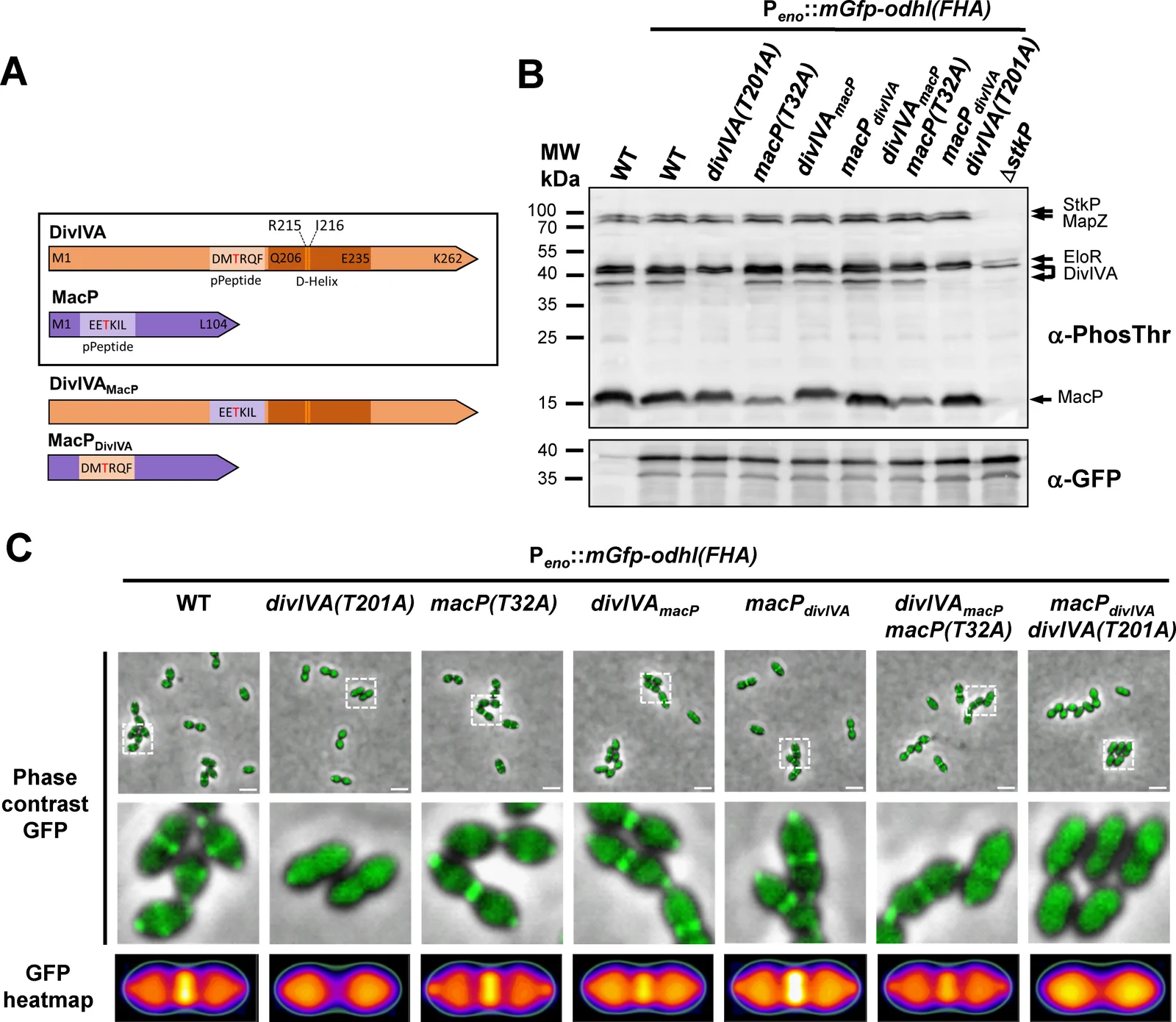
Modulating interactions between OdhI(FHA) and phosphorylated substrates (**A**) Schematic illustration of the phospho-swapping mutations between *divIVA* and *macP*. DivIVA’s phosphopeptide is DM-pT(201)-RQF; MacP’s phosphopeptide is EE-pT(32)-KIL; D-Helix corresponds to Q206–E235 helix in DivIVA; the two orange bands in D-Helix are R215–I216, mutated to G215–A216 in the strain P*_eno_::mGfp-odhI(FHA*), divIVA-Helix* in Fig. 5. (**B**) Western immunoblots of whole-cell lysates from phospho-swapped mutants expressing P*_eno_::mGfp-OdhI(FHA*), grown to exponential phase. Extracts were probed with anti-phospho-threonine (α-PhosThr, top panel) and anti-GFP (α-GFP, bottom panel) antibodies. Arrows highlight the phosphorylation signals for the different StkP substrates. (**C**) Representative-phase contrast/fluorescence overlays of exponential-phase cultures of phospho-swapped mutants expressing P*_eno_::mGfp-OdhI(FHA*); scale bar, 2 µm. A region of interest, marked with a dashed line, has been magnified for detailed visualization. Corresponding heatmaps representing the localization pattern of mGFP-OdhI(FHA) are shown on the bottom; from a total of 430 cells analyzed per strain, only early constricting cells were selected for illustration purposes. Results are representative of independent experiments performed three times.

The specific interaction between mGFP-OdhI(FHA) and the phosphorylated form of DivIVA led us to investigate its underlying molecular basis. It has been shown that the nature of the amino acid at the pThr +3 position is critical (59). In the mycobacterial GarA and corynebacterial OdhI homologs, the phospho-threonine peptide (ETT^P^SVF) recognized by their cognate FHA domain features a phenylalanine at the pThr +3 position (42, 54). Given that DivIVA also carries a phenylalanine at the pThr +3 position (DMT^P^RQF), unlike EloR, MacP, and MapZ, it was reasonable to hypothesize that this residue contributes to the interaction between phosphorylated DivIVA and OdhI’s FHA domain. To test this hypothesis, we swapped the phosphopeptide regions involved in interactions with FHA domains (positions −2 to +3 relative to the pThr) (59) between DivIVA and MacP (Fig. 4A). We reasoned that this swap would disrupt DivIVA’s recognition by OdhI’s FHA domain but would make phosphorylated MacP able to bind the sensor. We checked that both DivIVA with the MacP phosphopeptide (DivIVA_MacP_) and, conversely, MacP with the DivIVA phosphopeptide (MacP_DivIVA_) were efficiently phosphorylated by StkP *in vivo* (Fig. 4B). Surprisingly, DivIVA_MacP_ still interacted efficiently with the OdhI FHA domain, as mGFP-OdhI(FHA) still localized at the septum and at the poles, albeit with reduced fluorescence intensity at midcell (Fig. 4C). Similarly, MacP_DivIVA_ did not interact with the sensor (Fig. 4C). These observations suggest that the specificity of OdhI’s FHA domain for phosphorylated DivIVA cannot be attributed solely to the phosphopeptide sequence, and that other factors, such as the structural context or additional residues outside the swapped region, likely contribute to the specificity of the interaction.

**Fig 5:**
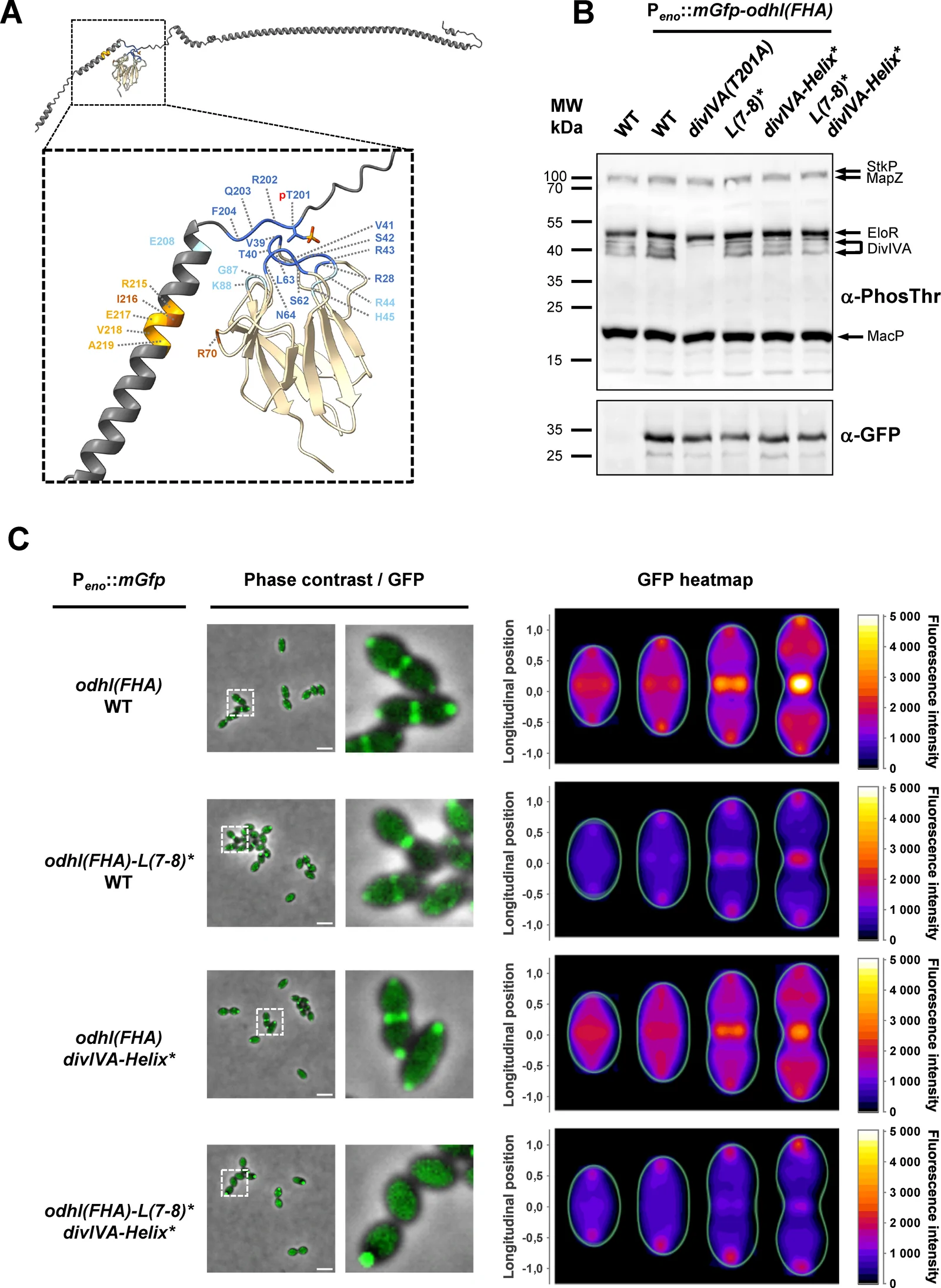
Structural and functional analysis OdhI(FHA)-phosphorylated DivIVA interaction(**A**) Complex between phosphorylated DivIVA and OdhI(FHA) predicted by AlphaFold3 (60). The complex is displayed in cartoon, and pThr in atoms. DivIVA is shown in dark gray, and OdhI(FHA) in light yellow. Regions in dark and light blue indicate residues from the first interaction interface detected with AlphaBridge (83) in Fig. S6B, with default and low confidence levels, respectively. Regions in dark and light orange indicate residues from the second interaction interface detected with AlphaBridge (83) in Fig. S6B, with default and low confidence levels, respectively. Details of the interfaces detected are summarized in Table S3. (**B**) Western immunoblots of whole-cell lysates from OdhI(FHA)/DivIVA interface mutants expressing P*_eno_::mGfp-OdhI(FHA*), grown to exponential phase. Extracts were probed with anti-phospho-threonine (α-PhosThr, top panel) and anti-GFP (α-GFP, bottom panel) antibodies. Arrows highlight the phosphorylation signals for the different StkP substrates. (**C**) Representative phase-contrast/fluorescence overlays of exponential-phase cultures of OdhI(FHA)/DivIVA interface mutants expressing P*_eno_::mGfp-OdhI(FHA*); scale bar, 2 µm. A region of interest, marked with a dashed line, has been magnified for detailed visualization. Corresponding heatmaps representing the localization pattern of mGFP-OdhI(FHA) are shown on the right; 850 cells were analyzed per strain. Note the presence of intense fluorescent foci in some P_eno_::mGfp-odhI(FHA)-L(7–8)* cells, likely reflecting protein aggregates, which may account for the global decrease observed in fluorescence intensity, yet the reduction remains particularly pronounced at the septum. Results are representative of independent experiments performed three times.

To further investigate this, we used AlphaFold3 (60) to predict the binding interfaces between the OdhI’s FHA domain and the phosphorylated forms of the four StkP substrates DivIVA, MacP, EloR, and MapZ (Fig. S6A). Predictions indicated that the OdhI’s FHA domain engages with all four phosphorylated substrates with comparable moderate confidence, primarily through a shared interface involving some of the canonical phospho-binding loops of the FHA domains (L4–5 and L6–7 loops) (61) and the different phosphopeptides (Fig. S6B). However, AlphaFold3 also revealed a unique potential secondary interaction specific to DivIVA. This additional interface was formed between the residues R215–A219 present in the α-helix (Q206-E235) located immediately downstream of DivIVA’s phosphopeptide and the residue R70 present in the L7-8 loop (N69-R70) of OdhI’s FHA domain (Fig. S6B; Fig. 5A).

To test whether this secondary contact might contribute to the specific recognition of phosphorylated DivIVA by OdhI’s FHA domain, we introduced mutations in either DivIVA’s α-helix (R215–A219, RIEVA to GAEVA, leading to P*_eno_::mGfp-odhI(FHA*), divIVA-Helix*), or OdhI’s FHA L7–8 loop (NR to QH as in EmbR(FHA) L7–8 loop that did not bind phosphorylated DivIVA (Fig. 1)), leading to P_eno_::mGfp-odhI(FHA)-L (7, 8)*, or both. After confirming that these mutations did not affect the phosphorylation profiles of the strains and the expression of mGFP-OdhI(FHA) (Fig. 5B), we assessed their ability to interact by analyzing the localization of the FHA sensor. Strikingly, mutating either region individually decreased the fluorescence signal observed at the division septum, reflecting that these regions are required for the interaction between OdhI’s FHA domain and phosphorylated DivIVA (Fig. 5C). Consistently, mutating both regions further decreased septal fluorescence (Fig. 5C). These findings challenge the current view about FHA domain specificity and suggest that additional, phosphorylation-independent contacts play a critical role in the interaction, thereby contributing to the specific recognition of a phosphopeptide by an FHA domain.

Overall, these results indicate that the specificity of the interaction does not rely only on a single interaction surface involving the phosphopeptide but rather arises from a combination of factors, including the broader structural context and residues outside the regions examined.

### Analysis of StkP and DivIVA dynamics

**Fig 6:**
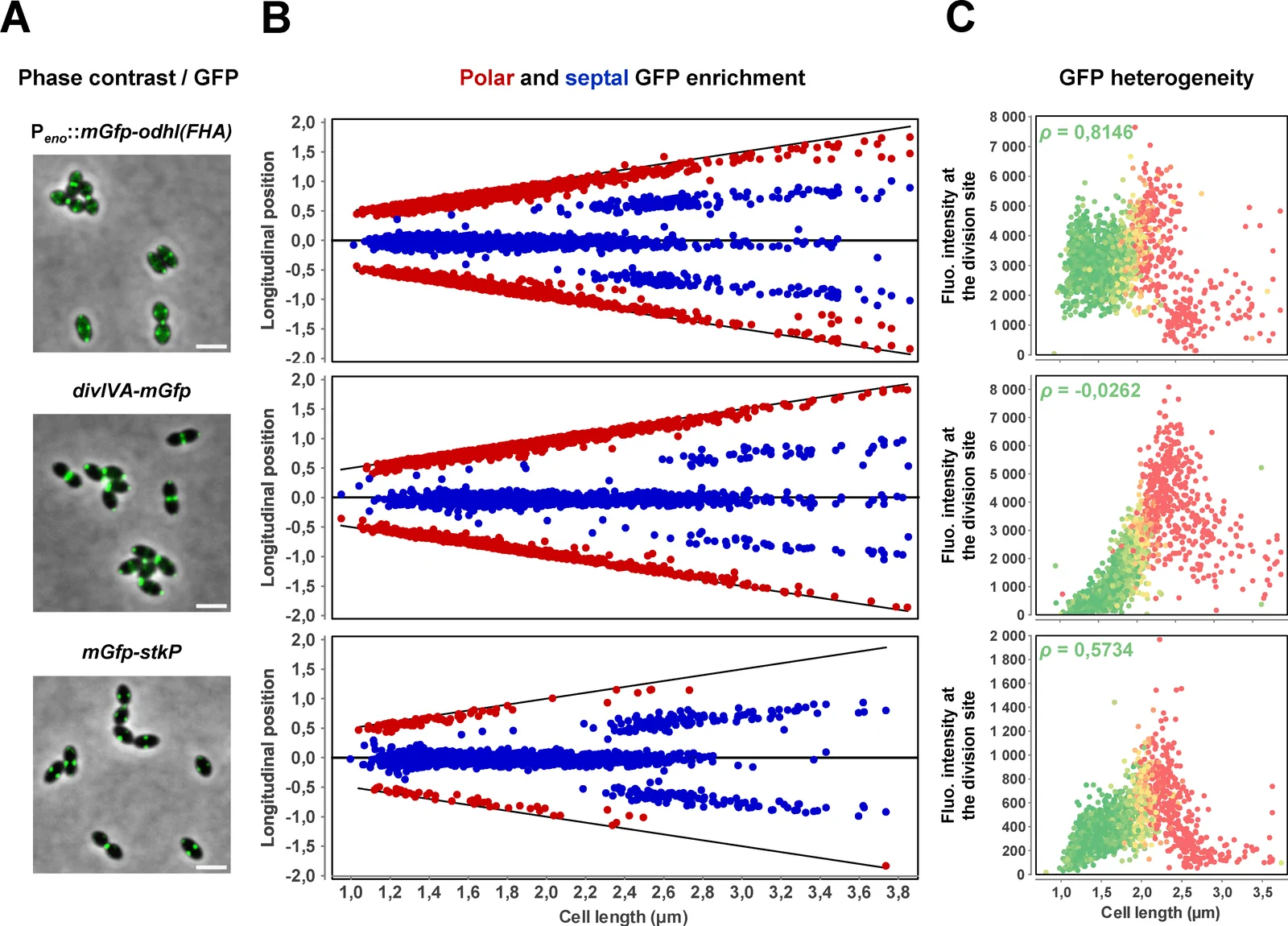
Dynamics of DivIVA phosphorylation (**A**) Representative phase-contrast/fluorescence overlays of exponential-phase cultures of WT and strains expressing P*_eno_::mGfp-OdhI(FHA*), *divIVA-mGfp,* and *mGfp-StkP*; scale bar, 2 µm. (**B**) Quantification of polar (red) and septal (blue) signals for mGFP-OdhI(FHA), DivIVA-mGFP, mGFP-StkP. Numbers and positions of the signals were plotted against cell length (µm). (**C**) Quantification of mean fluorescence intensity at the division site for mGFP-OdhI(FHA), DivIVA-mGFP, mGFP-StkP in individual cells and plotted against cell length (µm). Advancement of constriction is color-coded from green (newborn cells) to yellow (elongated cells), orange (constricting cells), and red (late-constricted cells). *P* = Spearman’s rank correlation coefficient; *P* was calculated between mean fluorescence intensity at the division site and cell length in non-constricted cells (constriction ratio < 1.04; dark green dots). Panels **A**, **B**, and **C** correspond to the same data set of 1,900 cells per strain. Results are representative of independent experiments performed three times.

To explore the dynamics of StkP-mediated protein phosphorylation at the single-cell level, we conducted a comparative analysis of the fluorescence patterns of the sensor mGFP-OdhI(FHA), the substrate DivIVA-mGFP and the kinase mGFP-StkP (Fig. 6). StkP and DivIVA are classified as late cell division proteins that relocate from the division septum of the mother cell (parental division site) to the cell equator (future division site of daughter cells) only at a very late stage of the cell division process (14) (Fig. 6A and B). As a result, mGFP-OdhI(FHA) was also expected to relocate at the cell equator at a late stage. Analysis of demographs reflecting the localization dynamics of the three proteins during the course of the cell cycle confirmed this prediction, as mGFP-StkP, DivIVA-mGFP, and mGFP-OdhI(FHA) largely exhibited similar localization patterns (Fig. 6A and B). However, careful inspection of mGFP-OdhI(FHA) dynamics showed that it appeared at the cell equator before DivIVA-mGFP (Fig. 6B). This suggests a potential additional interaction of the sensor with a yet unidentified phosphorylated partner.

Despite this difference, the similarity observed between the three fluorescence patterns suggested that StkP is active at all stages of the cell cycle, both at the parental division septum and the cell equators of daughter cells. However, one cannot exclude that phosphorylated forms of DivIVA could persist from one generation to the other. Indeed, even if the cognate StkP phosphatase PhpP has been shown to dephosphorylate DivIVA (14, 22), no evidence supports that it mediates its complete dephosphorylation *in vivo*. In this context, the localization of mGFP-OdhI(FHA) at the daughter cell equator may reflect not only the phosphorylation of DivIVA upon colocalization with StkP at the last stage of cell division, but also the relocalization of phosphorylated DivIVA from the parental division septum to the equator of daughter cells or from the previous cell cycle. To distinguish between these possibilities, we performed short pulses of *stkP* expression with the strain P*_comX_::stkP*_Δ*stkP*_P*_eno_::mGfp-OdhI(FHA*) before microscopy analyses (Fig. S7A and B). This strategy allowed us to assess StkP activity at any point in the cell cycle, obviating the potential carryover of the phosphorylated forms of DivIVA from the prior cell cycle. Under these conditions, we observed that mGFP-OdhI(FHA) localizes in a similar pattern to that seen with normal expression of *stkP* (Fig. S6A and B; Fig. 1C and 2B). This confirms that the signal of mGFP-OdhI(FHA) can be used as a readout of StkP activity and, consequently, that StkP is active throughout the cell cycle whenever localized at the division septum or at the cell equator of daughter cells.

### Localization of phosphorylated DivIVA at the poles

We noted that mGFP-OdhI(FHA) maintained polar localization consistently throughout the cell cycle. This observation raised two possibilities: either mGFP-OdhI(FHA) persists at the new poles after completion of cell constriction, or it actively relocalizes at the opposite old poles. To test these two scenarios, which are not mutually exclusive, we conducted time-lapse microscopy with the strain expressing P*_eno_::mGfp-OdhI(FHA)* (Fig. S7C). Interestingly, while mGFP-OdhI(FHA) persists at the new poles after completion of cell constriction as expected (purple stars), we, however, observed that it also relocates at old poles (orange stars). This observation is consistent with the presence of the polar signals of DivIVA detected in some cells even when StkP is only expressed briefly (Fig. S7A and B). It demonstrates that phosphorylated DivIVA at the pole is inherited not only from the completion of the parental division septum closure, but also from DivIVA dynamically relocates to the opposite pole inherited from the mother cell. Since StkP localizes at the division septum and the cell equators, where it logically phosphorylates its substrates, these findings suggest that phosphorylated DivIVA can localize *de novo* at the older pole. Consequently, they also suggest that the poles inherited from the mother cells are not inert and inactive places in *S. pneumoniae* as originally thought (27).

Finally, we examined the localization dynamics of mGFP-StkP, DivIVA-mGFP, and mGFP-OdhI(FHA) by comparing their midcell fluorescence intensities as a function of the cell length (Fig. 6C). This analysis revealed distinct fluorescence patterns among the three proteins, especially in newborn cells (Fig. 6C). For both DivIVA-mGFP and mGFP-StkP, midcell intensity steadily increased as a function of the cell length, indicating that their levels rise as the cell cycle progresses. However, mGFP-OdhI(FHA) showed high cell-to-cell variability in intensity in non-constricted cells. This suggested that, although StkP remains active through the cell cycle, its activity varies significantly from cell-to-cell in early stages of cell division. Because many promoters are intrinsically noisy, we tested mGFP alone under the control of the same enolase promoter (P*_eno_::mGfp*) as mGFP-OdhI(FHA) to ensure that the observed variability was not simply due to differences in sensor expression (Fig. S7A and B). We assessed fluorescence heterogeneity for the two fusions by using the maximum fluorescence intensity in cells as a proxy for signal enrichment and the coefficient of variation defined as the ratio of the standard deviation to the mean to quantify the variability between individual cells. Our analysis revealed that the cell-to-cell heterogeneity of mGFP-OdhI(FHA) in non-constricted cells was higher than what could be explained by the intrinsic promoter noise alone. This shows that the observed variability does not simply reflect differences in sensor expression but represents genuine differences in StkP-mediated protein phosphorylation between newborn cells.

## DISCUSSION

In this study, we developed a novel fluorescent sensor for StkP by exploiting the FHA domain of OdhI, which appears to interact with the phosphorylated form of DivIVA, a key substrate of StkP in *S. pneumoniae*. With this sensor, we analyzed StkP activity at the single-cell level, revealing that StkP is active whenever correctly localized at the septum throughout the cell cycle, but with a marked cell-to-cell heterogeneity, particularly in newly born cells. These observations point to differences in the activation of the kinase itself as the source of heterogeneity, highlighting how little we still know about the triggers of StkP activity. It is known that *stkP* mutants display defective cell constriction and division (13), whereas cells devoid of its cognate phosphatase PhpP are poorly viable (13, 62, 63). Therefore, one can speculate that StkP-mediated phosphorylation should be finely regulated in newborn cells, thereby ensuring the successful progression of cell division. Such an apparent heterogeneity of StkP activity in newborn cells is surprising and may result from an origin other than StkP activity. Alternatively, an explanation could relate to the characteristics of DivIVA itself. For example, DivIVA might be the less efficiently dephosphorylated substrate of PhpP phosphatase (62, 64). On the other hand, DivIVA could also be the most accessible substrate of StkP in the cell. In either case, phosphorylated DivIVA would thus outcompete the other phosphoproteins regarding the amount of phospho-threonine available for FHA domains and be the preferential target of FHA domains. This would be consistent with our observation that FhaA and FhaB FHA domains also interact with phosphorylated DivIVA (Fig. S4). Finally, it cannot be excluded that steric hindrance at the division site may contribute to heterogeneity in DivIVA phosphorylation, rather than in StkP activity itself.

The use of this sensor also raises exciting questions about the function of DivIVA at the poles, as mGFP-OdhI(FHA) localization at these sites highlights the presence of phosphorylated DivIVA. In *S. pneumoniae*, cell elongation does not occur through lateral peptidoglycan insertion like rod-shaped bacteria (65). Instead, the divisome assembles peptidoglycan for cell elongation from the cell center, thereby building the entire new cell half and pole of each daughter cell, while the other half and pole are inherited from the mother cell. The “old poles” are thus not actively involved in growth and division and are considered as inert regions, accumulating misfolded and aggregated proteins (27). Hence, and contrary to other species such as *M. tuberculosis* and *C. glutamicum* that exhibit polar growth (66), the persistence of DivIVA localization at the new pole could be seen as a relic of the divisome from the previous cell cycle. This is consistent with previous findings showing that DivIVA is more concentrated at the pneumococcal division site than the poles (28), suggesting that its role would be predominantly to promote uniform septum maturation. Moreover, DivIVA has the property to recognize the negative membrane curvatures (67). Consistently, it would thus remain localized on either side of the constriction septum until the formation of the new pole generated during cell division (27, 28). However, the fact that we observed the appearance of mGFP-OdhI(FHA) at the opposite poles, which is inherited from the mother cell, indicates that phosphorylated DivIVA can redistribute from the division septum to the old poles (Fig. S7D). This dynamic redistribution of DivIVA within cells is consistent with similar observations made in *B. subtilis*, where septal DivIVA contributes to polar DivIVA and inversely (68).

While DivIVA phosphorylation is key in regulating pneumococcal cell elongation (13, 52), it is unclear what the role of such phosphorylated proteins at the old poles would be. Interestingly, DivIVA phosphorylation is a conserved regulatory mechanism across bacteria, albeit on different residues and regions. However, the functional consequences of its phosphorylation vary depending on the organism. For instance, phosphorylation modulates DivIVA’s protein interaction network in *Mycobacterium smegmatis*. Indeed, phosphorylation regulates DivIVA’s binding to ParA and PapM, which are involved in chromosome segregation (69). Similarly, DivIVA phosphorylation is required for the interaction with the cell wall hydrolase MltG and to promote its localization at the division septum in *Streptococcus suis* (70). In *Streptococcus mutans*, DivIVA phosphorylation suppresses the essentiality of the cell division paralog *gpsB*, which regulates the StkP homolog PknB, thereby suggesting that altered DivIVA phosphorylation is toxic for the cell (71). In other bacteria, phosphorylation primarily affects DivIVA’s localization and function. Indeed, it modulates DivIVA’s localization dynamics, impacting polar growth and the establishment of cell polarity in *Deinococcus radiodurans* and *S. coelicolor* (72, 73). In *Mycobacteria*, phosphorylation of Wag31, the homolog of DivIVA, has been shown to stimulate its oligomerization and polar localization to promote the synthesis of polar peptidoglycan (9, 74). Moreover, it has been shown that phosphorylation favors high-order assembly of DivIVA/Wag31 at the pole, with the formation of more compact polymeric structures (75). Collectively, all these examples illustrate that DivIVA phosphorylation is critical to fine-tune DivIVA function in several aspects of the bacterial cell cycle. *In S. pneumoniae*, cells form chains with abnormally flattened (oblate) poles in the absence of DivIVA, thus reflecting improper daughter cell separation (26, 27, 52). Even though no major peptidoglycan synthesis and remodeling enzymes have been detected at the old poles, this absence does not exclude low-level or transient activities that remain below current detection limits. In this context, the presence of phosphorylated DivIVA at the old poles could be critical to maintain the correct curvature shaping the poles. This would be consistent with our observations (Fig. S5) (13) showing altered morphology and bulging phenotypes in the phosphoablative mutant of DivIVA. Future investigations are now needed to properly assess how DivIVA phosphorylation shapes the poles and regulates morphogenesis in *S. pneumoniae*.

Interestingly, we also observed that mGFP-OdhI(FHA) localization preceded shortly DivIVA-mGFP (Fig. 6B). This finding was unexpected, given our prior results demonstrating that mGFP-OdhI(FHA) specifically associates with the phosphorylated form of DivIVA. These observations imply that mGFP-OdhI(FHA) might transiently interact with an additional, yet unidentified, potentially phosphorylated partner in wild-type cells. However, in strains in which DivIVA is non-phosphorylatable, this alternative interaction is not detected, and the DivIVA phosphoablative mutation alone was sufficient to disrupt the septal localization of mGFP-OdhI(FHA) (Fig. 3A and B). This suggests that the interaction between mGFP-OdhI(FHA) and this putative additional partner is dependent on DivIVA phosphorylation. Interestingly, one possible candidate for this additional partner could be the DivIVA paralog GpsB, which positively regulates StkP activity in *S. pneumoniae* (52, 58) and PrkC in *B. subtilis* (76). Recently, GpsB has been shown to be phosphorylated in *S. pneumoniae*, and this phosphorylation is crucial in regulating StkP activity (58). It is therefore tempting to speculate that GpsB phosphorylation state could, in turn, be influenced by DivIVA phosphorylation itself. Such a model is plausible, as DivIVA and GpsB are part of a tightly interconnected regulatory network with StkP (52). Another possibility could be StkP itself since the timing of StkP localization at the future division sites coincides with that of mGFP-OdhI(FHA). Further investigations will therefore be required to determine whether GpsB, StkP, or another protein contributes to the early localization pattern of mGFP-OdhI(FHA).

Beyond these cellular and regulatory aspects uncovered with the use of the mGFP-OdhI(FHA) sensor, this work also raises broader questions about the specificity of recognition of FHA domains. The unique structure of FHA domains is primarily composed of 11 β-strands organized into two main β-sheets (36). Together, these sheets form a twisted β-sandwich core, stabilized by hydrophobic interactions (77). Although FHA domains vary in sequence, this fold is highly conserved due to the hydrophobic core provided by the β strands. Therefore, structural variation across FHA domains is mainly observed in the flexible loops connecting the β strands, which vary greatly in length and structural organization. Binding to phospho-threonine (pThr) peptides generally involves residues in the loops between the β-strand pairs β4–β5, β6–β7, and β10–β11 (61). While pThr peptide recognition has been linked to the amino acid at the pThr + 3 position (59), our observations do not support that the recognition mode is limited to this sequence feature (Fig. 4). Supporting this, we also observed that phosphorylated DivIVA is recognized by FhaA and FhaB FHA domains (Fig. S4), which do not share the same phosphopeptide preferences. This suggested that additional determinants, distinct from the phosphopeptide and the canonical recognition β-loops (61), may contribute to the specificity of the interaction. This hypothesis is supported by our observation that a secondary interaction surface lies between the L7–8 loop of OdhI(FHA) and the residue R208 present in the helix following the DivIVA phosphopeptide (Fig. 5 and Fig. S6).

Exploring the structural features of FHA domain interactions with pThr peptides promises to open exciting avenues for synthetic biology. By pinpointing variations in these structural components, we could potentially engineer FHA domains with tailored recognition capabilities, allowing them to selectively bind specific phosphorylated proteins in particular species. Gaining access to the phosphorylation status in single cells would thus provide crucial and novel insights into the field of bacterial serine/threonine phosphorylation.

## MATERIALS AND METHODS

### General procedures

*S. pneumoniae* strains were derived from R800 *rpsL1* (resistant to streptomycin). Strains were grown in C + Y medium or Todd-Hewitt yeast broth (Difco) at 37°C without agitation for liquid cultures and embedded in THY agar supplemented with 3% horse blood (TCS Biosciences) for growth on plates. For induction of the pComX promoter, chemically synthesized ComS peptide was used (concentrations and time of induction were indicated in figure legends when necessary) (56).

### Growth measurements

Growth assays were performed automatically at 37°C by reading optical density (550 nm) every 10 min after a brief shaking, with a Tecan SUNRISE microtiter plate reader. Cells were first grown in C + Y medium up to exponential phase, back-diluted to OD_550_ = 0.001, and transferred to a 96-well plate (Nunc Edge, nontreated and flat-bottom wells), whose lid was treated with 0.05% Triton-X100 and 20% ethanol to avoid condensation.

### Allelic replacement mutagenesis

Gene deletion or replacement was achieved through a two-step procedure based on a bicistronic *kan-rpsL* cassette (78). PCR products of interest were transformed as previously described, using competent cells incubated with synthetic competence-stimulating peptide 1 (CSP1) to induce competence (79). The strains used in this study and their construction are detailed in Table S1. The associated oligonucleotides are listed in Table S2. All strains were validated through colony PCR, antibiotic sensitivity assays, and locus sequencing.

### Microscopy and image analysis

Cells were grown in C + Y medium up to OD_550_ = 0.1–0.2, spotted onto C + Y agarose pad (1%), and covered with a cover glass. Slides were visualized using a Nikon TiE microscope, equipped with the perfect focus system (PFS, Nikon), and fitted with an Orca-CMOS Flash4 V2 camera with a 100 × 1.45 objective. Time-lapse microscopy was performed at 30°C through a thermostated chamber. Images were collected using NIS-Elements (Nikon), processed with Fiji software (80), and the MicrobeJ plugin (81). In MicrobeJ: background correction of the images was performed before analysis; the Feature/DivisionSite was used to quantify the specific fluorescence intensity at the division site in single cells; in Fig. 1, fluorescence kurtosis values were directly computed by MicrobeJ; in Fig. 6, the number of septal and polar signals were detected using the Feature/strip and Feature/spot, respectively. Statistical analyses in Fig. 6 and Fig. S7 were performed using GraphPad Prism 10 software (https://www.graphpad.com/).

### Preparation of crude extracts and immunoblot analysis

Cultures were grown in C + Y medium up to OD_550_ = 0.3–0.4, pelleted, and resuspended in TE buffer (Tris-HCl 10 mM pH 8, EDTA 1 mM), supplemented with a cocktail of protease inhibitors [CLAPA (Chymostatine 1 µg/mL, Leupeptine 1 µg/mL, Antipaine 1 µg/mL, Pepstatine 1 µg/mL, Aprotinine 5 µg/mL, Sigma-Aldrich) and phosphatase inhibitor cocktail 2 (Sigma-Aldrich)]. After cell disruption by sonication, 20 µg of crude extracts were analyzed by SDS-PAGE and electrotransferred onto an Immobilon-P membrane (Merck-Millipore). All primary antibodies were all rabbit antibodies and were used as follows: anti-GFP (Amsbio) 1/10,000 in TBST-BSA 1%; anti-phospho-threonine (Cell Signaling) 1/2,000 in TBST-BSA 5%; anti-StkP-PASTA 1/175,000 (13) in TBST-BSA 5%; anti-StkP-KD (13) 1/5,000 in TBST-BSA 5%. A goat anti-rabbit secondary antibody HRP conjugate (Bio-Rad) was used at 1/5,000 in TBST-BSA 1%. The signal was revealed using SuperSignal West Pico PLUS Chemiluminescent Substrate kit (Thermo Scientific), and the membranes were visualized using Fusion Fx (Vilber Lourmat).

### Structural predictions

Structural predictions of complexes between OdhI(FHA) and StkP-phosphorylated substrates were performed with AlphaFold3 (60). Complexes were inspected with PAE-Viewer (82) and AlphaBridge (83) for PAE matrices and interface representation, respectively. Model visualization and representation in Fig. 5 was performed using UCSF ChimeraX software (84).

## Data Availability

Data are available from the corresponding author upon request.

## SUPPLEMENTAL MATERIAL

Supplemental material mbio.01648-26-s0001.pdf Figures S1 to S7 and Tables S1 to S3.

ASM does not own the copyrights to Supplemental Material that may be linked to, or accessed through, an article. The authors have granted ASM a non-exclusive, world-wide license to publish the Supplemental Material files. Please contact the corresponding author directly for reuse.
