# Supplementary material for: Single-cell analysis of StkP activity reveals the temporal dynamics of DivIVA phosphorylation in Streptococcus pneumoniae
Source: mBio. 2026 Sep 21;17(10):e01648-26. doi: 10.1128/mbio.01648-26 (PMC13712964; doi:10.1128/mbio.01648-26)
Supplement: Supplementary file 1 [file mbio.01648-26-s0001.pdf]

SUPPLEMENTAL MATERIAL

Single cell analysis of StkP activity reveals the temporal dynamic of DivIVA phosphorylation in *Streptococcus pneumoniae*

Sylvie Manuse[1], Léo Claude[1], Pedro M Alzari[2], Anne Marie Wehenkel[3], Christophe Grangeasse[1,*]

[1]Molecular Microbiology and Structural Biochemistry (MMSB), UMR5086, CNRS, Université Lyon 1, Lyon, France

[2]Structural Microbiology Unit, Institut Pasteur, UMR3528, CNRS, Université Paris Cité, Paris, France

[3]Bacterial Cell Cycle Mechanisms Unit, Institut Pasteur, UMR3528, CNRS, Université Paris Cité, Paris, France

Figures S1 to S7

Tables S1 and S3

Supplemental references

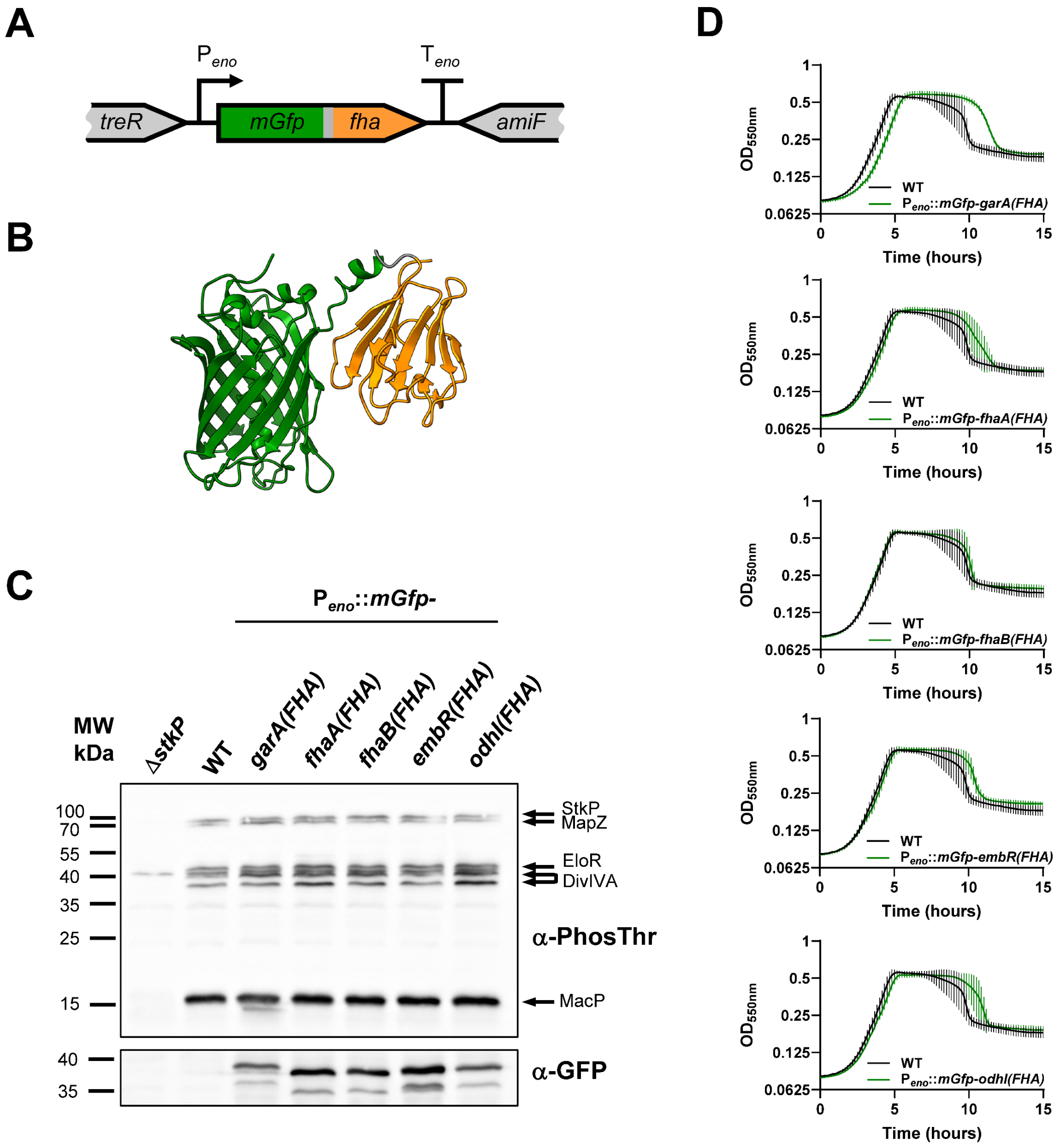

Figure S1: Construction of a sensor for StkP activity

(A) Schematic illustration of the genetically encoded FHA-based biosensors. Fusion genes encoding FHA domains fused to the C-terminus of mGFP were integrated into the genome of *S. pneumoniae*, at a neutral locus within the IS*1167* element between *treR* and *amiF*, and expressed under the constitutive promoter and terminator of the enolase gene. **(B)** AlphaFold2-based structural model of fluorescent FHA-based biosensors. **(C)** Western immunoblots of whole-cell lysates from the sensor strains, grown to exponential phase. Extracts from pneumococcal strains expressing the different FHA-based sensors were probed with anti-phosphothreonine (α-PhosThr, top panel) and anti-GFP (α-GFP, bottom panel) antibodies.

Arrows highlight the phosphorylation signals for the different StkP substrates. **(D)** Growth curves of the sensor strains. Pneumococcal strains expressing the different FHA-based sensors were grown in C+Y medium at 37°C. For each strain, data are the average results from 2 independent experiments performed with 3 biological replicates (*n* = 6). Error bars represent standard deviation. Data for the WT strain are the same across the different graphs.

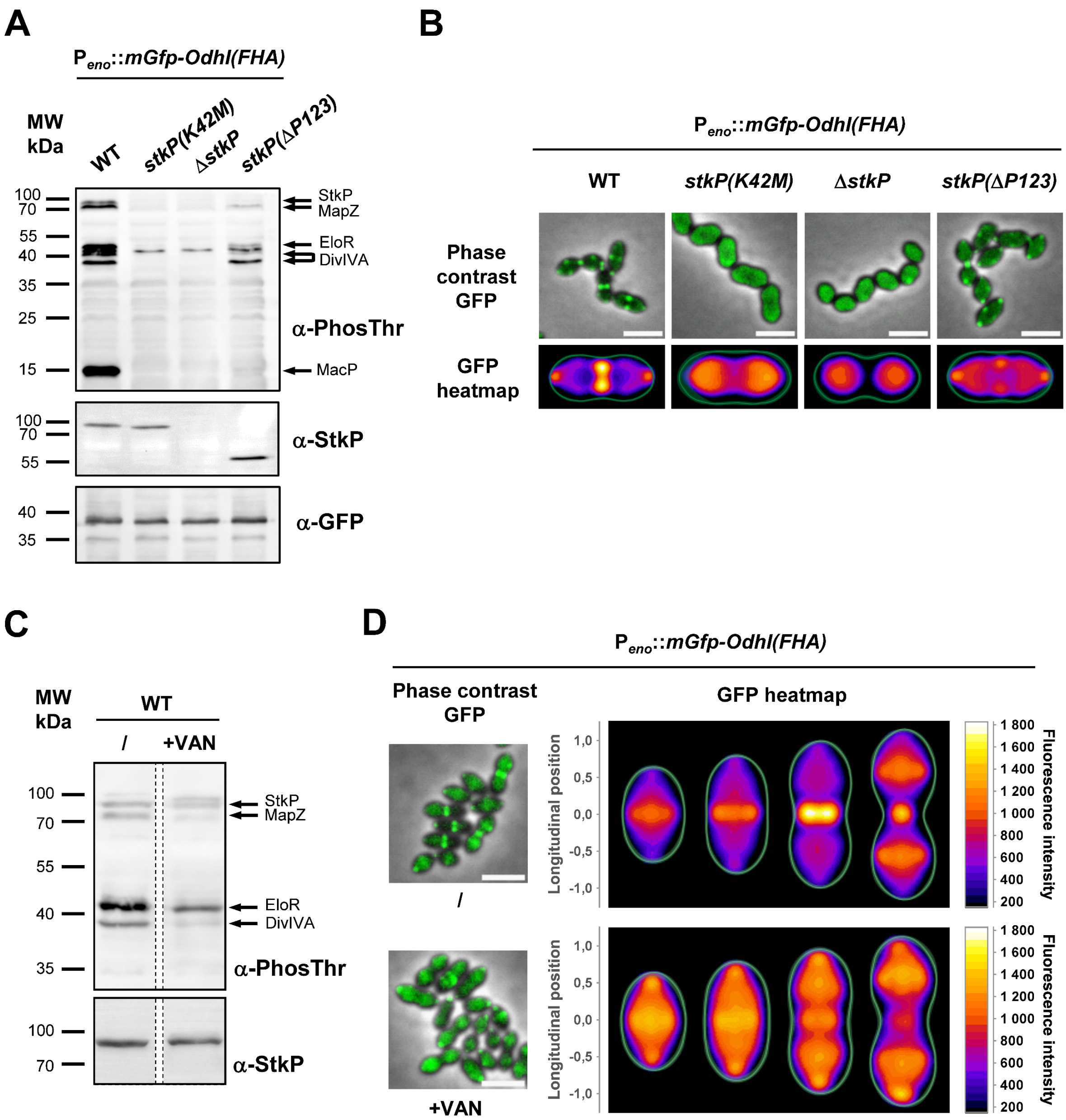

Figure S2: Differences in StkP activity highlighted by mGFP-OdhI(FHA)

(A) Western immunoblots of whole-cell lysates from WT and *stkP* mutants expressing P_{eno}::*mGfp-OdhI(FHA)*), grown to exponential phase. Extracts were probed with anti-phosphothreonine (α-PhosThr, top panel), anti-StkP (α-StkP, middle panel) and anti-GFP (α-GFP, bottom panel) antibodies. Arrows highlight the phosphorylation signals for the different StkP substrates. **(B)** Representative phase contrast/fluorescence overlays of exponential phase cultures of *stkP* mutants expressing P_{eno}::*mGfp-OdhI(FHA)*) before analysis in A); scale bar, 2 µm. Corresponding heatmaps representing the localization pattern of mGFP-OdhI(FHA) are

shown on the bottom; from a total of 400 cells analyzed per strain, only early constricting cells were selected for illustration purpose. Results are representative of independent experiments performed three times. **(C)** Western immunoblots of whole-cell lysates from WT strain grown to exponential phase, and treated or not with vancomycin (0.5 µg/mL for 30 min). Extracts were probed with anti-phosphothreonine (α-PhosThr, top panel) and anti-StkP (α-StkP, bottom panel) antibodies. Arrows highlight the phosphorylation signals for the different StkP substrates. **(D)** Representative phase contrast/fluorescence overlays of exponential phase cultures of WT strain expressing P_{eno}::*mGfp-OdhI(FHA)*) , treated or not with vancomycin (0.5 µg/mL for 30 min); scale bar, 2 µm. Corresponding heatmaps representing the localization pattern of mGFP-OdhI(FHA) are shown on the right, with a total of 800 cells analyzed per condition. Results are representative of independent experiments performed three times.

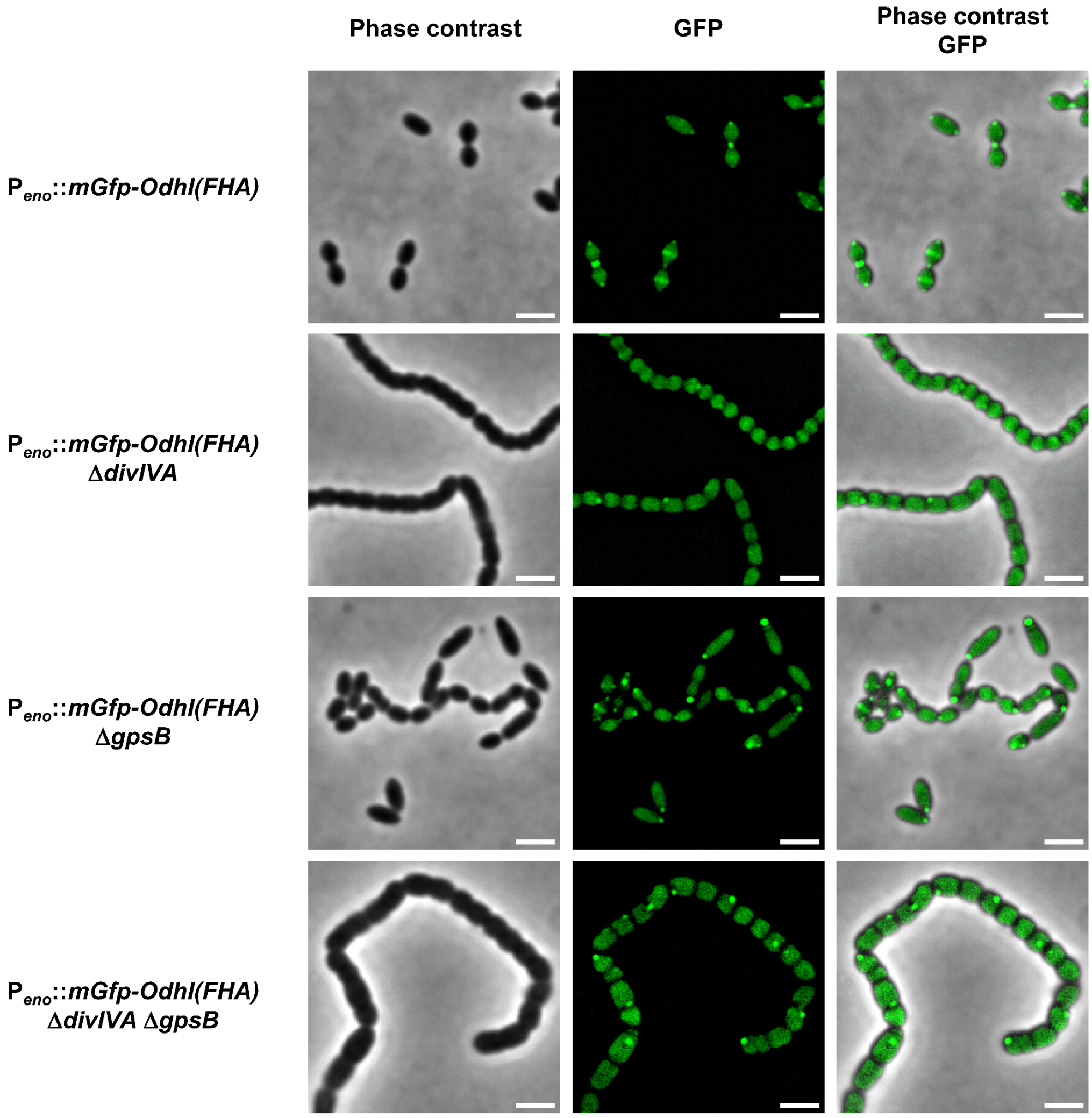

Figure S3: Impact of *divIVA* and *gpsB* deletion on mGFP-OdhI(FHA) signal

Representative images of exponential cultures of pneumococcal cells expressing P$_{eno}$::*mGfp-OdhI(FHA)*) in absence of *divIVA*, *gpsB*, or both. Phase contrast, fluorescent signal and overlays are shown; scale bar, 2 µm.

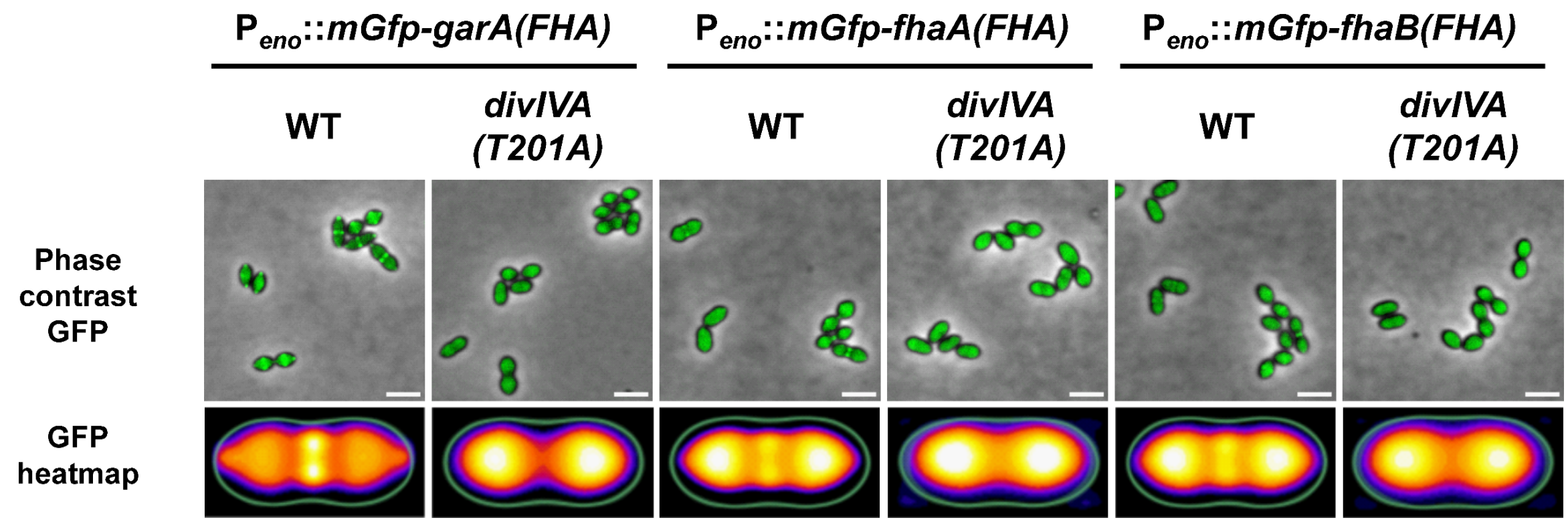

Figure S4: Interaction of multiple FHA domains with phosphorylated DivIVA

Representative phase contrast/fluorescence overlays of exponential phase cultures of WT and *divIVA(T201A)* strains expressing P_{eno}::*mGfp-OdhI(FHA)*); scale bar, 2 µm. Corresponding heatmaps representing the localization pattern of mGFP-OdhI(FHA) are shown on the bottom; from a total of 500 cells analyzed per strain, only early constricting cells were selected for illustration purpose. Results are representative of independent experiments performed three times.

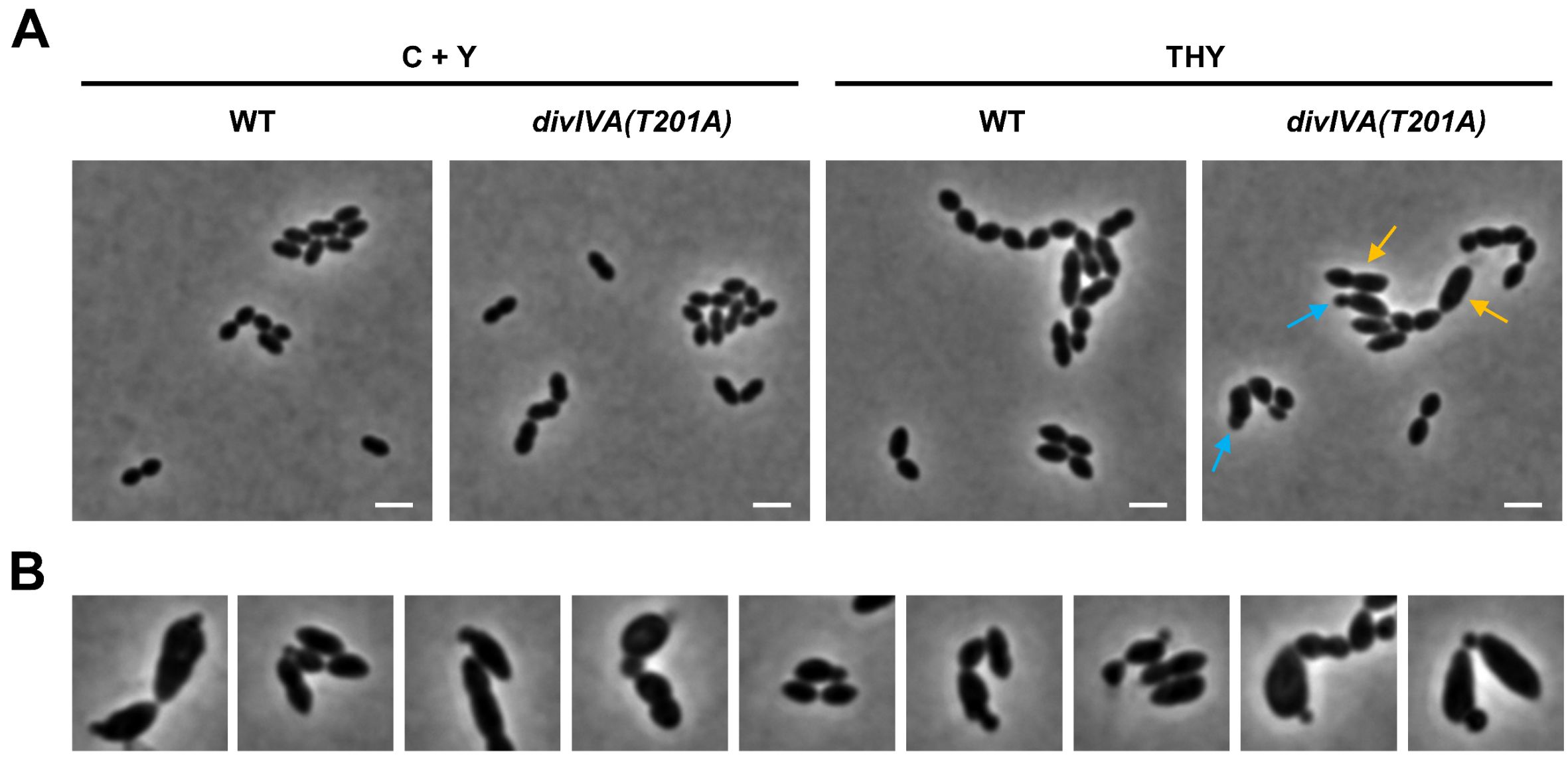

Figure S5: Impact of DivIVA phosphoablative mutation on cell morphology

(A) Representative phase contrast images of WT and *divIVA(T201A)* mutant, cultured in C + Y or THY media; scale bar, 2 µm. Blue arrows highlight polar bulging, and yellow arrows highlight elongated cells. **(B)** Representative selection of abnormal morphologies observed with the *divIVA(T201A)* mutant cultured in THY.

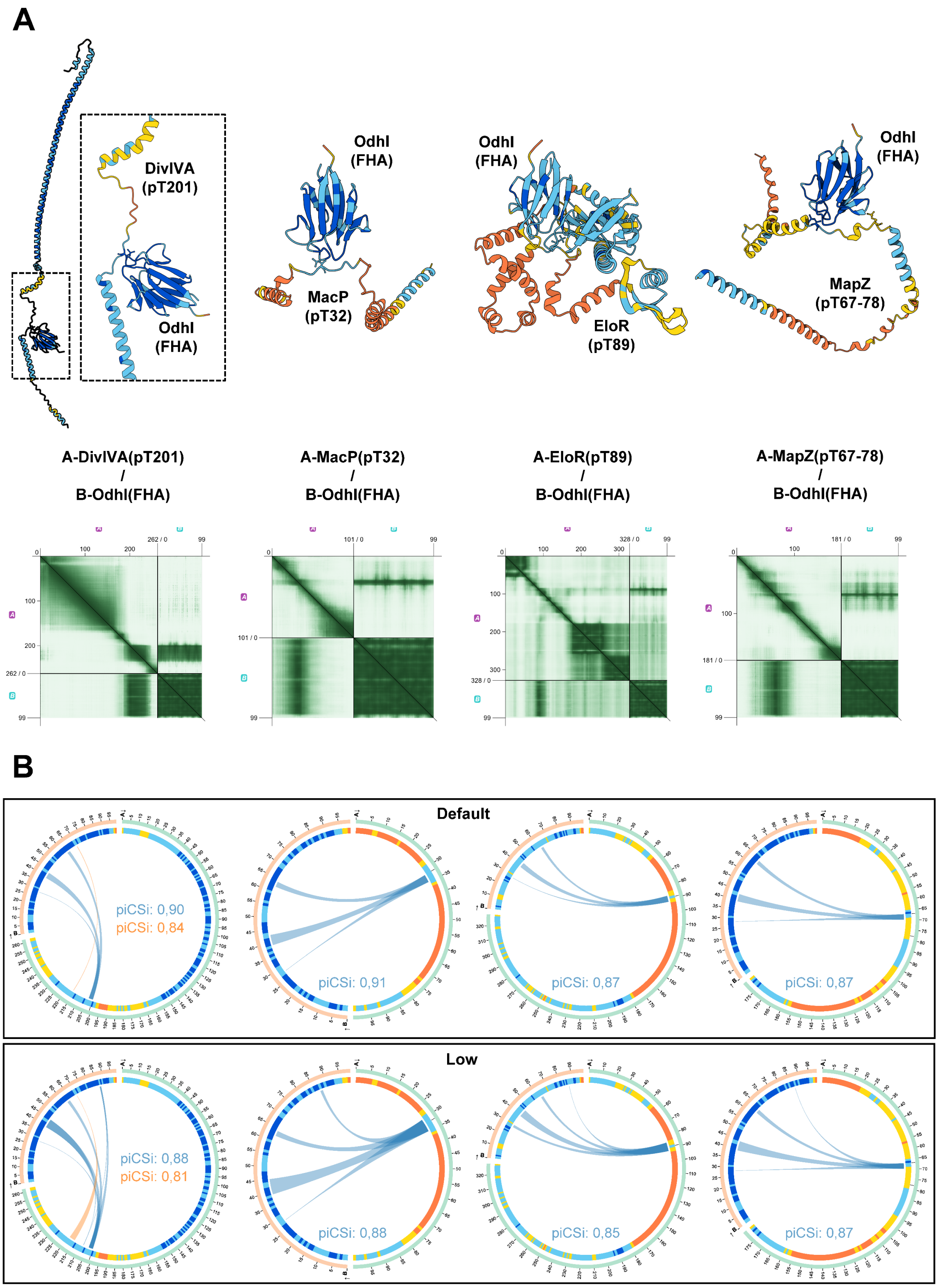

Figure S6: Structural prediction and interface analysis of OdhI(FHA) with phosphorylated substrates

(A) Complexes of the four StkP phosphorylated substrates [A] with OdhI(FHA) [B] predicted by AlphaFold3 (1). Residues are color-coded following pLDDT (predicted Local Distance

Difference) scores. PAE (Predicted Aligned Error) matrices visualized with PAE-Viewer (2) are shown below. For MacP and MapZ predictions, the proteins without their extracellular domain were used. Each substrate was predicted with their corresponding phospho-threonines (pT201 for DivIVA, pT32 for MacP, pT89 for EloR, pT67-78 for MapZ). **(B)** The AlphaBridge (3) plots corresponding to the predicted complexes are shown with default (top panel) and low (bottom panel) confidence levels for interface detection. piCSi (predicted Interaction Confidence Score) are indicated within the plots. Details of the interfaces detected are summarized in Table S3.

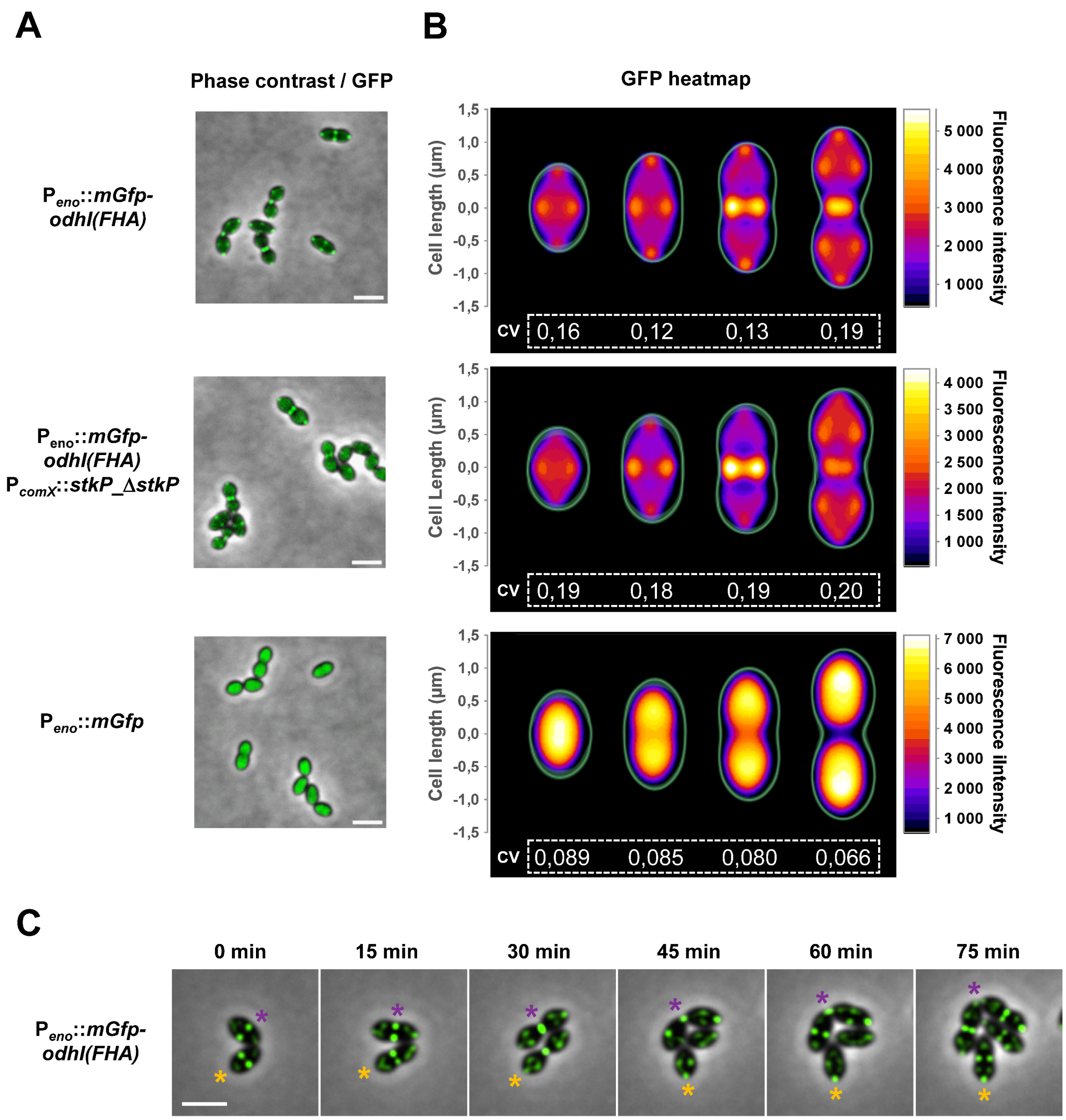

Figure S7: Localization and dynamics of mGFP-OdhI(FHA) in WT and *stkP* complemented strains

(A) Representative phase contrast/fluorescence overlays of exponential phase cultures of WT and P_{comX}::*stkP*_Δ*stkP* strains expressing P_{eno}::*mGfp-OdhI(FHA)*, and of WT strain expressing P_{eno}::*mGfp*; scale bar, 2 µm. In P_{comX}::*stkP*_Δ*stkP*_P_{eno}::*mGfp-OdhI(FHA)*, *stkP* expression was induced with 10 µM of ComS for 15 min right before observation. **(B)** Corresponding heatmaps representing the localization pattern of mGFP-OdhI(FHA) and mGFP are shown; *n* = 900, *n* = 600, *n* = 900 cells, respectively. CV = coefficient of variation; CV of maximum fluorescence

intensity was calculated for each cell cycle stage population and for each strain. Results are representative of independent experiments performed three times. **(C)** Time-lapse images (phase contrast/fluorescence overlays) of mGFP-OdhI(FHA) dynamics during cell growth and division; scale bar, 2 µm. Purple stars highlight formation of polar foci from division septum, orange stars highlight *de novo* formation of polar foci.

Table S1: Strain list

**Number**	**Strain**	**Genotype**	**Origin**	**Construction (primers listed in Table S2)**
NA	R800	R6 derivative	Gift from J.-P. Claverys (France)	
Spn5	WT	R800 *rpsL1*	Gift from J.-P. Claverys (France)	
Spn98	*stkP*::*kan-rpsL*	R800 *rpsL1*, *stkP*::*kan-rpsL*	(4)	
Spn10	Δ*stkP*	R800 *rpsL1*, Δ*stkP*	(4)	
Spn61	*stkP(K42M)*	R800 *rpsL1*, *stkP*::*stkP(K42M)*	(4)	
Spn751	*stkP(ΔP123)*	R800 *rpsL1*, *stkP*::*stkP(ΔP123)*	(5)	
Spn2259	*eloR*::*kan-rpsL*	R800 *rpsL1*, *eloR*::*kan-rpsL*	This study	PCR 3332-3333 (PCR 3332-3334#Spn5 + PCR 536-537#Spn98 + PCR 3335-3333#Spn5)
Spn3357	*eloR(T89A)*	R800 *rpsL1*, *eloR*::*eloR(T89A)*	This study	PCR 3332-3333 (PCR 3332-2300#Spn5 + PCR 2299-3333#Spn5)
Spn646	*divIVA*::*kan-rpsL*	R800 *rpsL1*, *divIVA*::*kan-rpsL*	(6)	
Spn90	*divIVA(T201A)*	R800 *rpsL1*, *divIVA*::*divIVA(T201A)*	(4)	
Spn267	Δ*divIVA*	R800 *rpsL1*, Δ*divIVA*	(6)	
Spn80	*gpsB*::*kan-rpsL*	R800 *rpsL1*, *gpsB*::*kan-rpsL*	(6)	
Spn86	Δ*gpsB*	R800 *rpsL1*, Δ*gpsB*	(6)	
Spn1268	*macP-kan-rpsL*	D39 *rpsL1*, Δ*cps*, *macP-kan-rpsL*	(7)	
Spn1273	*macP(T32A)*	D39 *rpsL1*, Δ*cps*, *macP*::*macP(T32A)*	(7)	
Spn190	*mapZ*::*kan-rpsL*	R800 *rpsL1*, *mapZ*::*kan-rpsL*	(8)	
Spn221	*mapZ(T67-78A)*	R800 *rpsL1*, *mapZ*::*mapZ(T67-78A)*	(8)	
Spn110	*mGfp-stkP*	R800 *rpsL1*, *stkP*::*mGfp-stkP*	(4)	
Spn130	*divIVA-mGfp*	R800 *rpsL1*, *divIVA*::*divIVA-mGfp*	(6)	

Spn3249	P_{eno}::*mGfp*	R800 *rpsL1*, IS*1167*::P_{eno}::*mGfp*	This study	PCR 946-947 (PCR 946-4073#Spn5 + PCR 4061-4062#Spn5 + PCR4169-4170#Spn110 + PCR 4063-4064#Spn5 + PCR 4074-947#Spn5)
Spn3574	P_{eno}::*mGfp-FHA(garA)*	R800 *rpsL1*, IS*1167*::P_{eno}::*mGfp-FHA(garA)*	This study	PCR 946-947 (PCR 946-4653#Spn3249 + PCR 4654-4072#gDNA(MTB) + PCR 4063-947#Spn3249)
Spn3579	P_{eno}::*mGfp-FHA(garA)*, *divIVA*::*kan-rpsL*	R800 *rpsL1*, IS*1167*::P_{eno}::*mGfp-FHA(garA)*, *divIVA*::*kan-rpsL*	This study	PCR 725-727#Spn646
Spn3580	P_{eno}::*mGfp-FHA(garA)*, *divIVA(T201A)*	R800 *rpsL1*, IS*1167*::P_{eno}::*mGfp-FHA(garA)*, *divIVA(T201A)*	This study	PCR 725-727#Spn90
Spn3575	P_{eno}::*mGfp-FHA(fhaA)*	R800 *rpsL1*, IS*1167*::P_{eno}::*mGfp-FHA(fhaA)*	This study	PCR 946-947 (PCR 946-4653#Spn3249 + PCR 4655-4656#gDNA(MTB) + PCR 4063-947#Spn3249)
Spn3581	P_{eno}::*mGfp-FHA(fhaA)*, *divIVA*::*kan-rpsL*	R800 *rpsL1*, IS*1167*::P_{eno}::*mGfp-FHA(fhaA)*, *divIVA*::*kan-rpsL*	This study	PCR 725-727#Spn646
Spn3582	P_{eno}::*mGfp-FHA(fhaA)*, *divIVA(T201A)*	R800 *rpsL1*, IS*1167*::P_{eno}::*mGfp-FHA(fhaA)*, *divIVA(T201A)*	This study	PCR 725-727#Spn90
Spn3576	P_{eno}::*mGfp-FHA(fhaB)*	R800 *rpsL1*, IS*1167*::P_{eno}::*mGfp-FHA(fhaB)*	This study	PCR 946-947 (PCR 946-4653#Spn3249 + PCR 4657-4658#gDNA(MTB) + PCR 4063-947#Spn3249)
Spn3583	P_{eno}::*mGfp-FHA(fhaB)*, *divIVA*::*kan-rpsL*	R800 *rpsL1*, *IS1167*::P_{eno}::*mGfp-FHA(fhaB)*, *divIVA*::*kan-rpsL*	This study	PCR 725-727#Spn646
Spn3584	P_{eno}::*mGfp-FHA(fhaB)*, *divIVA(T201A)*	R800 *rpsL1*, IS*1167*::P_{eno}::*mGfp-FHA(fhaB)*, *divIVA(T201A)*	This study	PCR 725-727#Spn90
Spn3577	P_{eno}::*mGfp-FHA(embR)*	R800 *rpsL1*, IS*1167*::P_{eno}::*mGfp-FHA(embR)*	This study	PCR 946-947 (PCR 946-4653#Spn3249 + PCR 4659-4660#gDNA(MTB) + PCR 4063-947#Spn3249)
Spn3578	P_{eno}::*mGfp-FHA(odhI)*	R800 *rpsL1*, IS*1167*::P_{eno}::*mGfp-FHA(odhI)*	This study	PCR 946-947 (PCR 946-4653#Spn3249 + PCR 4661-4662#gDNA(CGL) + PCR 4063-947#Spn3249)
Spn3671	P_{eno}::*mGfp-FHA(odhI)*, *eloR*::*kan-rpsL*	R800 *rpsL1*, IS*1167*::P_{eno}::*mGfp-FHA(odhI)*, *eloR*::*kan-rpsL*	This study	PCR 3332-3333#Spn2259

Spn3672	P_{eno}::*mGfp-FHA(odhI)*, *eloR(T89A)*	R800 *rpsL1*, IS*1167*::P_{eno}::*mGfp-FHA(odhI)*, *eloR(T89A)*	This study	PCR 3332-3333#Spn3357
Spn3585	P_{eno}::*mGfp-FHA(odhI)*, *divIVA*::*kan-rpsL*	R800 *rpsL1*, IS*1167*::P_{eno}::*mGfp-FHA(odhI)*, *divIVA*::*kan-rpsL*	This study	PCR 725-727#Spn646
Spn3586	P_{eno}::*mGfp-FHA(odhI)*, *divIVA(T201A)*	R800 *rpsL1*, IS*1167*::P_{eno}::*mGfp-FHA(odhI)*, *divIVA(T201A)*	This study	PCR 725-727#Spn90
Spn3673	P_{eno}::*mGfp-FHA(odhI)*, *macP-kan-rpsL*	R800 *rpsL1*, IS*1167*::P_{eno}::*mGfp-FHA(odhI)*, *macP-kan-rpsL*	This study	PCR 1202-1205#Spn1268
Spn3674	P_{eno}::*mGfp-FHA(odhI)*, *macP(T32A)*	R800 *rpsL1*, IS*1167*::P_{eno}::*mGfp-FHA(odhI)*, *macP(T32A)*	This study	PCR 1202-1205#Spn1273
Spn3675	P_{eno}::*mGfp-FHA(odhI)*, *mapZ*::*kan-rpsL*	R800 *rpsL1*, IS*1167*::P_{eno}::*mGfp-FHA(odhI)*, *mapZ*::*kan-rpsL*	This study	PCR 1037-1039#Spn190
Spn3676	P_{eno}::*mGfp-FHA(odhI)*, *mapZ(T67-78A)*	R800 *rpsL1*, IS*1167*::P_{eno}::*mGfp-FHA(odhI)*, *mapZ(T67-78A)*	This study	PCR 1037-1039#Spn221
Spn4239	P_{eno}::*mGfp-FHA(odhI)*, *stkP*::*kan-rpsL*	R800 *rpsL1*, IS*1167*::P_{eno}::*mGfp-FHA(odhI)*, *stkP*::*kan-rpsL*	This study	PCR 538-541#Spn98
Spn4240	P_{eno}::*mGfp-FHA(odhI)*, Δ*stkP*	R800 *rpsL1*, IS*1167*::P_{eno}::*mGfp-FHA(odhI)*, Δ*stkP*	This study	PCR 538-541#Spn10
Spn4241	P_{eno}::*mGfp-FHA(odhI)*, *stkP(K42M)*	R800 *rpsL1*, IS*1167*::P_{eno}::*mGfp-FHA(odhI)*, *stkP(K42M)*	This study	PCR 538-541#Spn61
Spn4242	P_{eno}::*mGfp-FHA(odhI)*, *stkP(ΔP123)*	R800 *rpsL1*, IS*1167*::P_{eno}::*mGfp-FHA(odhI)*, *stkP(ΔP123)*	This study	PCR 538-541#Spn751
Spn3800	P_{comX}::*stkP*	R800 *rpsL1*, *bgaA*::P_{comR}::*comR*, cpsO-N::P_{comX}::*stkP*	This study	PCR 1946-1943 (PCR 1946-2081#Spn1797 + PCR 4743-4176#Spn5 + PCR 2082-1943#Spn1797)
Spn3801	P_{comX}::*stkP*, *stkP*::*kan-rpsL*	R800 *rpsL1*, *bgaA*::P_{comR}::*comR*, cpsO-N::P_{comX}::*stkP*, *stkP*::*kan-rpsL*	This study	PCR 538-541#Spn98
Spn3802	P_{comX}::*stkP*, Δ*stkP*	R800 *rpsL1*, *bgaA*::P_{comR}::*comR*, cpsO-N::P_{comX}::*stkP*, Δ*stkP*	This study	PCR 538-541#Spn10

Spn3976	P_{comX}::*stkP*, Δ*stkP*, *IS1167*::*kan-rpsL*	R800 *rpsL1*, *bgaA*::P_{comR}::*comR*, cpsO-N::P_{comX}::*stkP*, Δ*stkP*, IS*1167*::*kan-rpsL*	This study	PCR 946-947#Spn1001
Spn3977	P_{comX}::*stkP*, Δ*stkP*, P_{eno}::*mGfp-FHA(odhI)*	R800 *rpsL1*, *bgaA*::P_{comR}::*comR*, cpsO-N::P_{comX}::*stkP*, Δ*stkP*, IS*1167*::P_{eno}::*mGfp-FHA(odhI)*	This study	PCR 946-947#Spn3578
Spn4503	P_{eno}::*mGfp-FHA(odhI)*, $divIVA_{macP}$	R800 *rpsL1*, IS*1167*::P_{eno}::*mGfp-FHA(odhI)*, $divIVA_{macP}$	This study	PCR 725-727 (PCR 725-5405#Spn5 + PCR 5406/727#Spn5)
Spn4504	P_{eno}::*mGfp-FHA(odhI)*, $divIVA_{macP}$, *macP-kan-rpsL*	R800 *rpsL1*, IS*1167*::P_{eno}::*mGfp-FHA(odhI)*, $divIVA_{macP}$, *macP-kan-rpsL*	This study	PCR 1202-1205#Spn1268
Spn4505	P_{eno}::*mGfp-FHA(odhI)*, $divIVA_{macP}$, *macP(T32A)*	R800 *rpsL1*, IS*1167*::P_{eno}::*mGfp-FHA(odhI)*, $divIVA_{macP}$, *macP(T32A)*	This study	PCR 1202-1205#Spn1273
Spn4506	P_{eno}::*mGfp-FHA(odhI)*, $macP_{divIVA}$	R800 *rpsL1*, IS*1167*::P_{eno}::*mGfp-FHA(odhI)*, $macP_{divIVA}$	This study	PCR 1202-1205 (PCR 1202-5403#Spn5 + PCR 5404/1205#Spn5)
Spn4507	P_{eno}::*mGfp-FHA(odhI)*, $macP_{divIVA}$, *divIVA*::*kan-rpsL*	R800 *rpsL1*, IS*1167*::P_{eno}::*mGfp-FHA(odhI)*, $macP_{divIVA}$, *divIVA*::*kan-rpsL*	This study	PCR 725-727#Spn646
Spn4508	P_{eno}::*mGfp-FHA(odhI)*, $macP_{divIVA}$, *divIVA(T201A)*	R800 *rpsL1*, IS*1167*::P_{eno}::*mGfp-FHA(odhI)*, $macP_{divIVA}$, *divIVA(T201A)*	This study	PCR 725-727#Spn90
Spn4620	P_{eno}::*mGfp-FHA(odhI)-L(7-8)**	R800 *rpsL1*, IS*1167*::P_{eno}::*mGfp-FHA(odhI)-L(7-8)**	This study	PCR 946-947 (PCR 946-5556#Spn3578 + PCR 5557-947#Spn3578)
Spn4622	P_{eno}::*mGfp-FHA(odhI)*, *divIVA-Helix**	R800 *rpsL1*, IS*1167*::P_{eno}::*mGfp-FHA(odhI)*, *divIVA-Helix**	This study	PCR 725-727 (PCR 725-5563#Spn5 + PCR 5564-727#Spn5)
Spn4865	P_{eno}::*mGfp-FHA(odhI)-L(7-8)**, *divIVA*::*kan-rpsL*	R800 *rpsL1*, IS*1167*::P_{eno}::*mGfp-FHA(odhI)-L(7-8)**, *divIVA*::*kan-rpsL*	This study	PCR 725-727#Spn646
Spn4866	P_{eno}::*mGfp-FHA(odhI)-L(7-8)**, *divIVA-Helix**	R800 *rpsL1*, IS*1167*::P_{eno}::*mGfp-FHA(odhI)-L(7-8)**, *divIVA-Helix**	This study	PCR 725-727#Spn4622
Spn4910	P_{eno}::*mGfp-FHA(odhI)*, Δ*divIVA*	R800 *rpsL1*, IS*1167*::P_{eno}::*mGfp-FHA(odhI)*, Δ*divIVA*	This study	PCR 725-727#Spn267

Spn4911	P_{eno}::*mGfp-FHA(odhI)*, *gpsB*::*kan-rpsL*	R800 *rpsL1*, IS*1167*::P_{eno}::*mGfp-FHA(odhI)*, *gpsB*::*kan-rpsL*	This study	PCR 815-818#Spn80
Spn4915	P_{eno}::*mGfp-FHA(odhI)*, Δ*gpsB*	R800 *rpsL1*, IS*1167*::P_{eno}::*mGfp-FHA(odhI)*, Δ*gpsB*	This study	PCR 815-818#Spn86
Spn4912	P_{eno}::*mGfp-FHA(odhI)*, Δ*divIVA*, *gpsB*::*kan-rpsL*	R800 *rpsL1*, IS*1167*::P_{eno}::*mGfp-FHA(odhI)*, Δ*divIVA*, *gpsB*::*kan-rpsL*	This study	PCR 815-818#Spn80
Spn4916	P_{eno}::*mGfp-FHA(odhI)*, Δ*divIVA*, Δ*gpsB*	R800 *rpsL1*, IS*1167*::P_{eno}::*mGfp-FHA(odhI)*, Δ*divIVA*, Δ*gpsB*	This study	PCR 815-818#Spn86

Table S2: Primer list

Number	Name	Sequence
538	SG74-phpP-up(-521)	GGTCAGGTTACTGCTCTGGAC
541	SG77-stkP-down-(+2483)	GAAAGGAGCACTAAAGGTCGC
725	Amont-divIVA-forward	TTCTGGCGATGATAGGATG
727	Aval-divIVA-reverse	CGTACTCACGGCAAAGTTTCAAG
815	SG148-spr0332-up-(-790)	GCCAAGCCCTGAGACAAATAG
818	SG151-spr0332-down-(+1088)	GGAACCGAGCTCCAAGTGG
946	SG182-treR-up-(0)	ATGAAGAAATACCAACAATTATTTAAGCAAATCC
947	SG183-amiF-up-(+486)	ACCCTCATGAATTCTCAGGCGGT
1037	Spr0334-amont-forward	GTCTAGCCTCTTTAAACGTGG
1039	Spr0334-aval-rev	GCATCAAGTCATAGCTTTCTGC
1202	JPL7-spr0893-sense-(-557)	ATGGAGTTTGAAGAAAAAACGCTTAGCC
1205	JPL10-spr0893-antisense-(+1024)	TTAATCTAAAGCCTTCAAAAAGGCC
2081	SG281-PcomXSther-(0)	TGAACCTCCAATAATAAATATAAAT
2082	SG282-cpsN-O-(+1106)	TTTCTAATATGTAACTCTTCCCAAT
3332	AP119-EloR-amont (+)	AAATGCGTAAAGTATTTAAAGAAATGGG
3333	AP120-EloR-aval (-)	CTCACCTTCTCGCGTAAAGTAC
3334	AP121-delor::K7 (-)	CATTATCCATTAAAAATCAAACGGTACCAGATTCCTCCTTATTTATTTCG
3335	AP122-delor::K7 (+)	AGGAAAGGGGCCCAGGTCTCTGTAAAATCAGGTTTATCCTGATTTTTTG

4061	up-eno-F	CCACCATGAGCTTTTTCAAG
4062	up-eno-R	CATTTTTTACTCTCCTTATGAG
4063	down-eno-F	AATAGTTCAGTGAACTATTTTATCC
4064	down-eno-R	CGTCACAAAGAACATGAAAG
4072	garA-FHA-R	GGATAAAATAGTTCACTGAACTATTTTACGGGCCCCCGGTACTCCCGTCATC
4073	up-IS1167-R	CTTGAAAAAGCTCATGGTGGCTCCATAATATCTATAAGGGATTTA
4074	down-IS1167-F	CTTTCATGTTCTTTGTGACGTCAAACTTATCAAGCGCAATGC
4169	const-GFP-spn-F	CTCATAAGGAGAGTAAAAAATGATTTCTAAAGGTGAAGAATTG
4170	const-GFP-spn-R	GGATAAAATAGTTCACTGAACTATTTTATTTATACAATTCATCCATAC
4176	pComx-endStkP-R(bis)	ATTGGGAAGAGTTACATATTAGAAATTAAGGAGTAGCTGAAGTTG
4653	linker(sensor)-mGFP-R	GTGCAACCCTTTATACAATTC
4654	FHA(garA)-F	GAATTGTATAAAGGGTTGCACGGCTCGGCGTTGCTGGTAGTC
4655	FHA(fhaA)-F	GAATTGTATAAAGGGTTGCACGCCGGAACGTCGGTTACGCTG
4656	FHA(fhaA)-R	GGATAAAATAGTTCACTGAACTATTTTAGTGCATGCGGACGATGATCTC
4657	FHA(fhaB)-F	GAATTGTATAAAGGGTTGCACGCTGCACGCTACCTGGTGGTG
4658	FHA(fhaB)-R	GGATAAAATAGTTCACTGAACTATTTTACGGGCGCAACTCGATTGCAG
4659	FHA(embR)-F	GAATTGTATAAAGGGTTGCACGGCCAGCAGGCGGTGGCCTAC
4660	FHA(embR)-R	GGATAAAATAGTTCACTGAACTATTTTACGTGCCGCCATGCGTCCCCGCGCTG
4661	FHA(odhI)-F	GAATTGTATAAAGGGTTGCACGGATCTGCTCTTCTTGTAGTC
4662	FHA(odhI)-R	GGATAAAATAGTTCACTGAACTATTTTACTCAGCAGGGCCTGCGAGGAAAACC
4743	pComX-stkP-F	TATTTATTATTGGAGGTTCAATGATCCAAATCGGCAAG
5403	macP-phosphoP(divIVA)-swap_R	GGAAGAAGAGGTTGGGAACTGACGTGTCATATCATTATCATCTAGCAAAG
5404	macP-phosphoP(divIVA)-swap_F	CTTTGCTAGATGATAATGATATGACACGTCAGTTCCCAACCTCTTCTTCC
5405	divIVA-phosphoP(macP)-swap_R	CATTTCTGCTTGAGATAAAATCTTAGTTTCCTCAATTGGTTCTTCTTC

5406	divIVA-phosphoP(macP)-swap_F	GAAGAAGAACCAATTGAGGAAACTAAGATTTTATCTCAAGCAGAAATG
5556	odhI(FHA)-L(7-8)embR_R	GAGCGTTGCGTGGCTCGTGCTGAACGTAGGTTCCGTTG
5557	odhI(FHA)-L(7-8)embR_F	CAACGGAACCTACGTTCAGCACGAGCCACGCAACGCTC
5563	divIVA-helix-GAEVA_R	CTTTATCGGCTACCTCAGCACCAGCTTGTAATTCTTCC
5564	divIVA-helix-GAEVA_F	GGAAGAATTACAAGCTGGTGCTGAGGTAGCCGATAAAG

Table S3: Interaction interface detection scores from AlphaBridge of OdhI(FHA) with phosphorylated substrates

Default confidence detection scores		
DivIVA(pT201) (A) - OdhI(FHA) (B)		
Interaction 1		
piCSi: 0,90		
Link	Sequence 1	Sequence 2
L1	A – 201-204	B – 28-28
L2	A – 201-204	B – 39-43
L3	A – 201-204	B – 62-64
Interaction 2		
piCSi: 0,84		
Link	Sequence 1	Sequence 2
L1	A – 216-216	B – 70-70
MacP(pT32) (A) - OdhI(FHA) (B)		
Interaction 1		
piCSi: 0,91		
L3	A – 32-35	B – 62-64
EloR(pT89) (A) - OdhI(FHA) (B)		
Interaction 1		
piCSi: 0,87		
Link	Sequence 1	Sequence 2
L1	A – 87-92	B – 28-28
L2	A – 87-92	B – 39-43
L3	A – 87-92	B – 62-64
MapZ(pT67-78) (A) - OdhI(FHA) (B)		
Interaction 1		
piCSi: 0,87		
Link	Sequence 1	Sequence 2
L1	A – 67-70	B – 28-28
L2	A – 67-70	B – 39-43
L3	A – 67-70	B – 62-64

Low confidence detection scores		
DivIVA(pT201) (A) - OdhI(FHA) (B)		
Interaction 1		
piCSi: 0,88		
Link	Sequence 1	Sequence 2
L1	A – 201-204	B – 28-28
L2	A – 201-204	B – 39-45
L3	A – 201-204	B – 62-64
L4	A – 201-204	B – 87-88
L5	A – 208-208	B – 39-45
L6	A – 208-208	B – 87-88
Interaction 2		
piCSi: 0,81		
Link	Sequence 1	Sequence 2
L1	A – 215-219	B – 70-70
MacP(pT32) (A) - OdhI(FHA) (B)		
Interaction 1		
piCSi: 0,88		
ink	Sequence 1	Sequence 2
L1	A – 30-35	B – 28-28
L2	A – 30-35	B – 39-45
L3	A – 30-35	B – 62-64
L4	A – 30-35	B – 87-88
EloR(pT89) (A) - OdhI(FHA) (B)		
Interaction 1		
piCSi: 0,85		
Link	Sequence 1	Sequence 2
L1	A – 85-92	B – 28-30
L2	A – 85-92	B – 39-45
L3	A – 85-92	B – 62-64
L4	A – 85-92	B – 87-87
MapZ(pT67-78) (A) - OdhI(FHA) (B)		
Interaction 1		
piCSi: 0,87		
Link	Sequence 1	Sequence 2
L1	A – 67-70	B – 28-28
L2	A – 67-70	B – 39-44
L3	A – 67-70	B – 62-64
L4	A – 67-70	B – 87-87

Supplemental references (also cited in the Main Text with different numbering)

1. Abramson J, Adler J, Dunger J, Evans R, Green T, Pritzel A, Ronneberger O, Willmore L, Ballard AJ, Bambrick J, Bodenstein SW, Evans DA, Hung C-C, O'Neill M, Reiman D, Tunyasuvunakool K, Wu Z, Žemgulytė A, Arvaniti E, Beattie C, Bertolli O, Bridgland A, Cherepanov A, Congreve M, Cowen-Rivers AI, Cowie A, Figurnov M, Fuchs FB, Gladman H, Jain R, Khan YA, Low CMR, Perlin K, Potapenko A, Savy P, Singh S, Stecula A, Thillaisundaram A, Tong C, Yakneen S, Zhong ED, Zielinski M, Žídek A, Bapst V, Kohli P, Jaderberg M, Hassabis D, Jumper JM. 2024. Accurate structure prediction of biomolecular interactions with AlphaFold 3. Nature 630:493–500.
2. Elfmann C, Stülke J. 2023. PAE viewer: a webserver for the interactive visualization of the predicted aligned error for multimer structure predictions and crosslinks. Nucleic Acids Research 51:W404–W410.
3. Álvarez-Salmoral D, Borza R, Maiella C, Kwee BPY, Xie R, Joosten RP, Hekkelman ML, Perrakis A. 2024. AlphaBridge: tools for the analysis of predicted biomolecular complexes doi:10.1101/2024.10.23.619601. openRxiv.
4. Fleurie A, Cluzel C, Guiral S, Freton C, Galisson F, Zanella-Cleon I, Di Guilmi AM, Grangeasse C. 2012. Mutational dissection of the S/T-kinase StkP reveals crucial roles in cell division of <i>Streptococcus pneumoniae</i>. Molecular Microbiology 83:746–758.
5. Zucchini L, Mercy C, Garcia PS, Cluzel C, Gueguen-Chaignon V, Galisson F, Freton C, Guiral S, Brochier-Armanet C, Gouet P, Grangeasse C. 2017. PASTA repeats of the protein kinase StkP interconnect cell constriction and separation of Streptococcus pneumoniae. Nature Microbiology 3:197–209.
6. Fleurie A, Manuse S, Zhao C, Campo N, Cluzel C, Lavergne J-P, Freton C, Combet C, Guiral S, Soufi B, Macek B, Kuru E, Vannieuwenhze MS, Brun YV, Di Guilmi A-M, Claverys J-P, Galinier A, Grangeasse C. 2014. Interplay of the Serine/Threonine-Kinase StkP and the Paralogs DivIVA and GpsB in Pneumococcal Cell Elongation and Division. PLoS Genetics 10:e1004275.
7. Fenton AK, Manuse S, Flores-Kim J, Garcia PS, Mercy C, Grangeasse C, Bernhardt TG, Rudner DZ. 2018. Phosphorylation-dependent activation of the cell wall synthase PBP2a in <i>Streptococcus pneumoniae</i> by MacP. Proceedings of the National Academy of Sciences 115:2812–2817.
8. Fleurie A, Lesterlin C, Manuse S, Zhao C, Cluzel C, Lavergne J-P, Franz-Wachtel M, Macek B, Combet C, Kuru E, Vannieuwenhze MS, Brun YV, Sherratt D, Grangeasse C. 2014. MapZ marks the division sites and positions FtsZ rings in Streptococcus pneumoniae. Nature 516:259–262.
